# Deciphering Ball Milling Mechanochemistry via Molecular Simulations of Collision‐Driven and Liquid‐Assisted Reactivity

**DOI:** 10.1002/anie.202505263

**Published:** 2025-10-23

**Authors:** Rupam Gayen, Leonarda Vugrin, Zehua Zhang, György Hantal, Ivan Halasz, Ana‐Sunčana Smith

**Affiliations:** ^1^ PULS Group, Department of Physics, Friedrich Alexander Universität Erlangen‐Nürnberg, IZNF Cauerstrasse 3 91058 Erlangen Germany; ^2^ Division of Physical Chemistry Ruđer Bošković Institute Bijenička c. 54 Zagreb 10163 Croatia

**Keywords:** Collision‐driven reactivity, Liquid‐assisted milling, Mechanochemistry, Molecular dynamics, Theoretical simulations

## Abstract

Mechanochemistry by ball milling proceeds through a series of discrete, high‐energy collisions between milling balls and the sample, yet the molecular‐level processes that govern the resulting chemical and physical transformations remain poorly understood. In this study, we develop a molecular dynamics simulation protocol to investigate a model mechanochemical reaction between potassium chloride (KCl) and 18‐crown‐6 ether, both under dry conditions and in the presence of water as a liquid additive. Our simulations reveal that the reaction is initiated by collision‐induced fragmentation of the KCl crystal into individual ions. This process occurs when the absorbed energy per ion pair during a collision exceeds the crystal's cohesion energy. We further show that the addition of a small amount of water facilitates the formation of complexes between potassium ions and 18‐crown‐6 molecules. However, excessive water content stabilizes the reactants instead, thereby suppressing complex formation. These findings highlight a non‐linear relationship between liquid additive concentration and the reaction outcome. Our approach offers a molecular‐level perspective on mechanochemical reactivity, providing valuable insights that could guide the rational optimization of milling conditions—particularly the targeted selection and dosing of liquid additives—to improve reaction efficiency.

## Graphical abstract

Supported by experiments, molecular dynamics simulations were developed to reveal how mechanochemical reactions, such as between KCl and 18‐crown‐6 with water as a liquid additive, proceed. Product formation is triggered by collision‐induced fragmentation, with liquid content showing a non‐linear effect. These insights clarify how collision energy and additives affect reactivity, aiding the optimization of milling conditions.

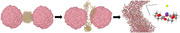

## Introduction

It is common in synthesis to purposefully initiate or accelerate chemical reactions by supplying energy in the form of heat, light, energy stored in a potential, or by delivering mechanical energy.^[^ ^1^ ^]^ Mechanical energy provided by collisions in a milling apparatus is particularly suitable to drive reactions of solid reagents and materials.^[^ ^2^, ^3^ ^]^ However, this technique resides only at the level of general understanding,^[^ ^4^, ^5^, ^6^, ^7^ ^]^ and the established connection between individual collisions and the bulk transformation^[^ ^8^, ^9^ ^]^ still does not provide a description of molecular‐level processes at the sites of collisions.

Mechanical processing provides activation of the milled material.^[^ ^10^, ^11^, ^12^, ^13^ ^]^ For example, the milled solid retains part of the energy that is dissipated during ball milling “tied up with structural and electronic defects.”^[^ ^14^ ^]^ This excess energy has been measured, and an energy threshold required to initiate a polymorph transformation has been established,^[^ ^15^ ^]^ in line with the finding that a specific amount of energy needs to be absorbed by the milled sample to advance the reaction by a certain extent.^[^ ^16^ ^]^

A milling process, however, is typically a result of numerous discrete high‐energy collisions. Each generates highly non‐equilibrium conditions that persist briefly and only in the impacted region.^[^ ^17^, ^18^ ^]^ Capturing events at the site of collision with chemical accuracy is a major experimental challenge, but one which could be addressed using theoretical modeling. So far, molecular dynamics (MD) simulations of indentation of two crystal particles,^[^ ^19^, ^20^, ^21^, ^22^ ^]^ demonstrated the appearance of defects and mixing of reactants at the molecular level,^[^ ^23^ ^]^ which was confirmed experimentally.^[^ ^24^ ^]^ Numerous other techniques have also been applied, particularly to study fracture dynamics.^[^ ^25^, ^26^ ^]^ Accounting for chemical transformations, under the kinetic effect of collision; however, has not been addressed.

Here, supported by experiments, we develop an approach based on non‐equilibrium molecular dynamics simulations to study chemical and physical processes in a single collision, with atomic resolution. We show that for the momenta and energies of the milling balls used in experiments, fragmentation of crystalline particles should be expected in order to enable a chemical reaction, which is followed by coarsening and formation of large agglomerates containing the product species. We also reveal conclusive evidence on the mechanistic behavior of water as a liquid additive, demonstrating a non‐linear efficiency in complex formation as a function of the amount of the liquid additive, due to competing solvation effects.

## Results and Discussion

### Model Reaction

In developing the simulations protocol, we have made certain stipulations for the model reaction to fulfill. It had to be such that it was indeed a mechanochemical reaction that required persistent high‐energy mechanical collision to advance. It should render molecular simulations easier if interactions between species could be captured by Coulombic effects, while avoiding the necessity to model breaking and making of covalent bonds. The reaction should also be sensitive to an additive in order to test if the simulations protocol could address the well‐known, but poorly understood, effect of the liquid additives in mechanochemistry.^[^ ^27^ ^]^ Finally, the course of the reaction should be resolved in a time‐dependent fashion.^[^ ^28^ ^]^

We have settled on the supramolecular encapsulation of $K^{+}$ by 18‐crown‐6 ether (18c6), using solid KCl (Figure S1 and Section S1). That the $K^{+}$ ion fits well in the cavity of an 18c6 molecule forming a stable supramolecular complex is well‐known^[^ ^29^ ^]^ and has been exploited to increase the basicity of KOH by stabilizing $K^{+}$.^[^ ^30^, ^31^ ^]^ Previously, when an effort had not been made to remove moisture from the reaction system, mechanochemical encapsulation of potassium cations proceeded readily, leading to the formation of the hydrated complex [(18c6K)]Cl×H_2_O.^[^ ^32^ ^]^ The reaction does not proceed with stirring of the reactants alone, with or without water additive, confirming that it is mechanochemical in nature (vide infra).

### Experimental Investigation of Dry Complexation of 18c6 with KCl

**Figure 1:**
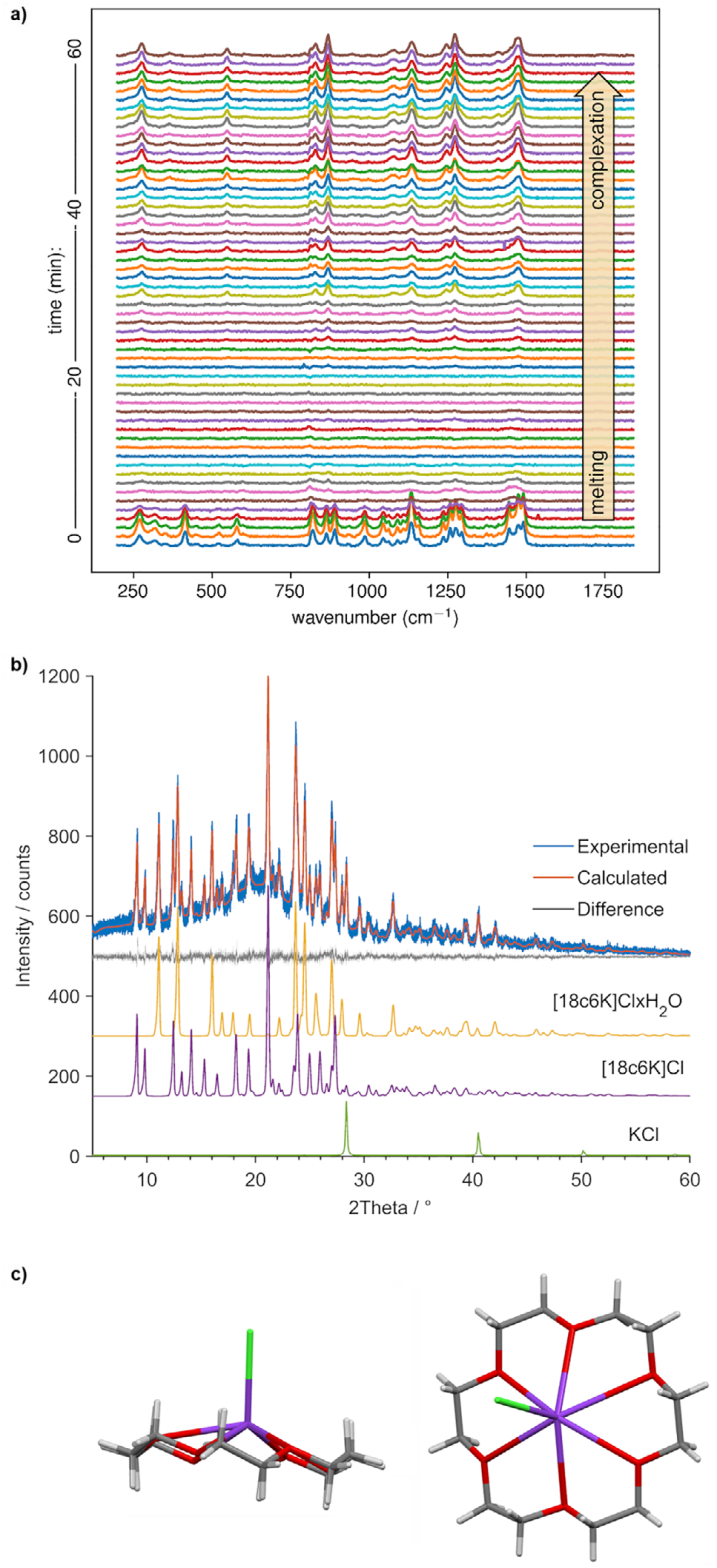
Experimental investigation of the mechanochemical reaction between KCl and 18c6 under dry‐nitrogen atmosphere. a) Time‐resolved Raman spectra of 18c6 complexation with KCl while heating at 50 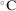 demonstrating initial melting of 18c6 followed by a steady formation of the complex. b) Rietveld refinement plot for the crystal structure solution and refinement of the anhydrous [(18c6)K]Cl complex. The sample had absorbed some moisture during handling and data collection leading to its partial transformation to the monohydrate resulting in a three‐component mixture. The individual contributions of each of the three phases (in yellow, purple and green) are provided beneath the difference curve. c) The molecular structure of the complex as determined by PXRD.

Our current experiments were performed by preparing the equimolar reaction mixture of 18c6 and KCl in a nitrogen‐filled glove box, in air‐tight PMMA milling vessels (Figures S2– S5). Milling using two stainless steel balls of 7 mm in diameter as the milling media and at room temperature under dry conditions provided no complexation reaction, as evident from the in situ collected Raman spectra, which did not change over the course of 90 min of milling (Figure S6). If however, the same experiment is repeated, but with the reaction mixture externally heated to 50 °C, which is above the melting point of 18c6, we first observe loss of Raman signal corresponding to melting of 18c6, followed by the steady appearance of new Raman bands indicating the formation of a new phase (Figure 1a and Figure S7).

The steady formation of the complex in the heated apparatus over the course of 60 min of ball milling indicates a process that depends on a persistent external stimulus, which is in this case the transfer of mechanical energy brought on by collisions with the milling balls. We confirmed that mechanochemical collisions are necessary for complexation by stirring dispersed powder of KCl in the melt of 18c6, which resulted in no complex formation (Figure S8).

Analysis of the milling product by powder X‐ray diffraction (PXRD) indicated the presence of the monohydrate complex,^[^ ^32^ ^]^ which appeared due to the absorption of moisture during transfer of the sample from the glove box and the powder diffractometer (see Section S1.4), a small amount of unreacted KCl and a hitherto unknown phase. After accounting for the contributions of the monohydrate and KCl in the measured PXRD pattern, the diffraction peaks belonging to the unknown phase were identified and indexed with an orthorhombic unit cell: a=11.623(2) Å, b=18.119(4) Å, c=8.249(2) Å, and V=1737.2(6) Å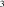. Crystal structure solution in the $P2_{1}2_{1}2_{1}$ space group was achieved by simulated annealing assuming the circular open conformation of the 18c6 molecule, and independent ions $K^{+}$ and ${\mathrm{Cl}}^{-}$. The solution, providing a good Rietveld fit to the diffraction pattern, revealed an anhydrous complex with $K^{+}$ in the cavity of 18c6 and with the chloride anion bonded (Figure 1b,c) (see Section S1.4).^[^ ^33^ ^]^ The new crystal structure was validated by periodic DFT calculations,^[^ ^34^ ^]^ which were initiated from the experimentally determined configuration. Following geometry optimization, only minor structural changes were observed, preserving the experimentally assigned space group and the characteristic tilted conformation of the chloride ligand. The absence of imaginary frequencies in the computed phonon dispersion confirms the dynamical stability of the optimized structure (for more information see Section S1.4).

### Establishing the Simulation Protocol: KCl Fragmentation

**Figure 2:**
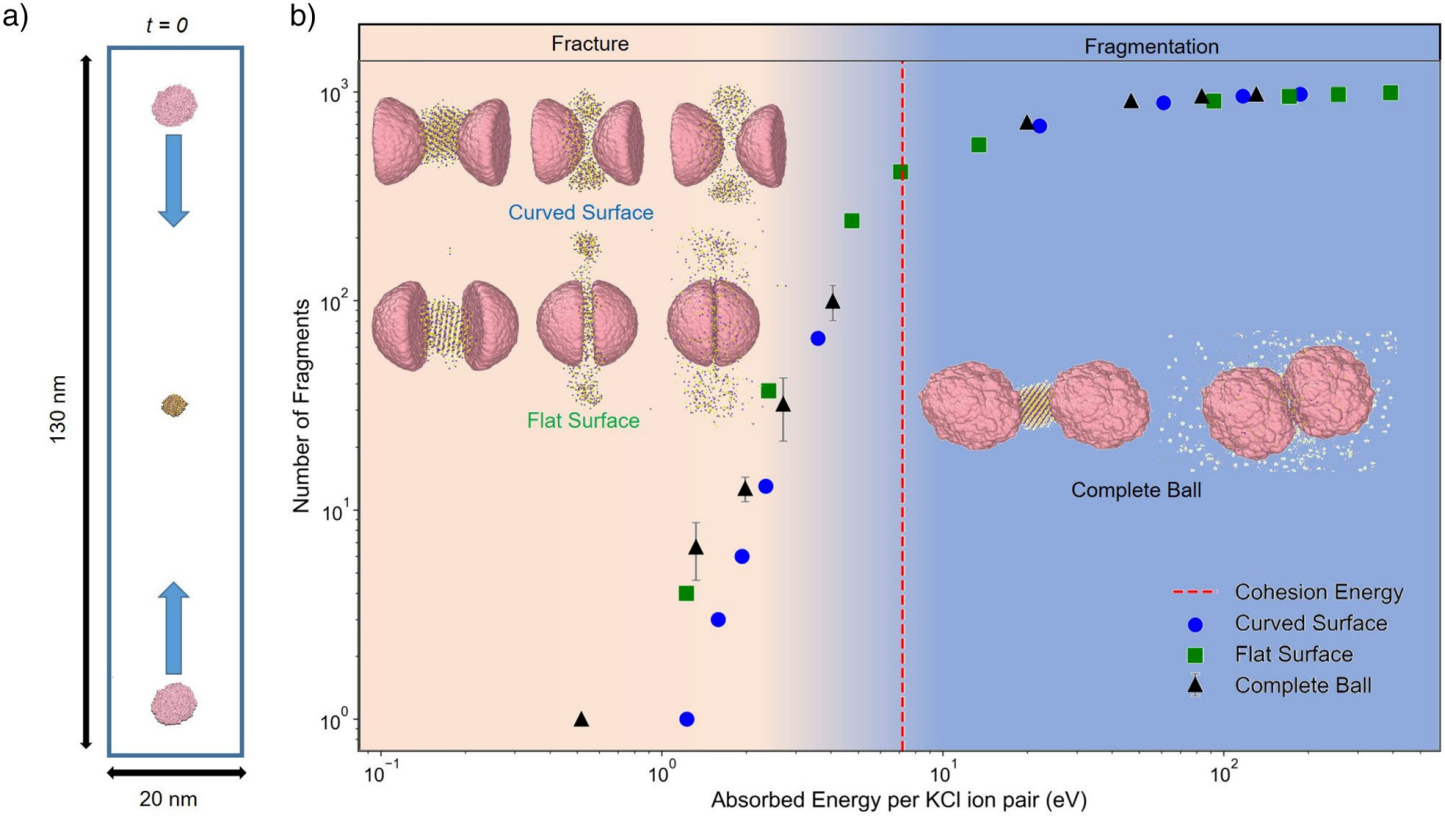
Simulation of a collision‐induced fragmentation and recrystallisation of a KCl crystal particle. a) Snapshot of the simulation at the beginning of the collision. The balls (in pink) are provided with equal and opposite centre‐of‐mass velocities in the range 0.1–10 nm ${\mathrm{ps}}^{-1}$ at 375 K to simultaneously collide with the KCl particle. b) Number of formed fragments as a function of absorbed energy in the collision for model milling balls of different curvature and stiffness. By a single fragment we consider a collection of ions where each ion has at least one neighbor within the distance of 3.9 Å (note that the KCl bond length in the crystal is 3.15 Å). The difference between the total energy after the collision and the total energy before the collision for KCl crystal provides the energy absorbed by the KCl crystal during the collision. The simulations were performed for three distinct crystal configurations of KCl and the results are the average of these three independent runs. Error bars were calculated as standard deviation of the mean.

To provide a detailed understanding of the observed processes, we conceived a protocol to simulate a collision event (for details, see Section S2), using molecular dynamics in the GROMACS 2021.5 software package.^[^ ^35^, ^36^ ^]^ Reactants ($K^{+}$, ${\mathrm{Cl}}^{-}$, 18c6), are modeled using the OPLS‐AA force field,^[^ ^37^ ^]^ in conjunction with the SPC/E $H_{2}O$,^[^ ^38^ ^]^ which was shown to correctly capture hydration of $K^{+}$ and ${\mathrm{Cl}}^{-}$.^[^ ^39^, ^40^ ^]^ All simulations were performed in triplicates starting with different KCl crystal particles all consisting of 500 ion pairs, spontaneously organized into a face‐centered cubic lattice (Section S2.3.1). The crystal particle was placed centrally between two milling balls in a rectangular box ($x\times y\times z=130\times 20\times 20\;{\text{nm}}^{3}$; Figure 2a). However, particular care had to be taken to the design of the grinding balls.

In both laboratory conditions and in simulations, the role of milling balls is to deliver in collisions its kinetic energy to reactants, causing fracture. In experiments, typically, with around one centimeter in size and weight in grams, they achieve kinetic energies of around 10 mJ—by far exceeding the energy in our molecular dynamics simulations. To capture the essence of these effects in simulations, we establish several design principles of milling balls (Section S2.2). In short, our in silico creations are amorphous structures with a mass of $3.5*10^{6}\;\text{amu}$, made up of 21 464 artificial neutral atoms, interacting by basically repulsive van der Waals potentials, defining the atom size and, accordingly, the chemical roughness. Neighboring atoms are connected by strong covalent bonds represented by harmonic springs that provide stiffness and integrity to hemispherical designs (23 Å radius), as well as to softer spherical (35 Å radius) balls. The balls may deform during the collision, but no shedding or topological change is permitted. By colliding the hemispheres with their curved or flat sides, as well as by using the softer balls, we explore the effects of stiffness, size and curvature of designs on the simulated milling process (Figure 2).

We find that the collision is characterized by the energy that the KCl particle absorbs per ion pair in the collision ($E_{a}$), which stems from the change in the kinetic and potential energies of all ions building the KCl crystal before collision and its fragments after the collision (Figure S18). Focusing the discussion on $E_{a}$ allows us to circumvent uncertainties in capturing the energy transfer in the impact associated with the design of the ball, and the choice of impact geometry. The absorbed energy is calculated following the protocol presented in Section S2.3.3. As expected, $E_{a}$ is associated with the gain in the of kinetic energies of the fragments, upon the release of the potential energy stored in the crystal. Immediately after the collision, this kinetic energy could be seen as increased local temperature or heat, which is gradually removed from the system by the reformation of the crystal and the dissipation to the environment through coupling with the thermostat.

Our findings (Figure 2b) indicate that the chemico‐physical properties of milling balls are significant only at low‐energy collisions, where the absorbed energy per ion pair is less than the cohesion energy of the crystal (for KCl this is $E_{c}$ = 7.19 eV).^[^ ^41^ ^]^ In this regime, the shape and chemical roughness of the balls influence the conversion of kinetic energy into mechanical excitation in a collision. This effect is particularly evident when examining the number of fragments to which a KCl crystal particle disintegrates, as a function of the initial kinetic energy of the balls (Figure S19). Although stiffer and less curved balls are more efficient in the transfer of kinetic energy, a general trend emerges: for low absorbed energies ($E_{a}$ < 0.5 eV), only vibrational modes in the KCl particle are excited, potentially leading to the formation of dislocations (Figure S20). At slightly higher absorbed energies (0.5 < $E_{a}$ < 2 eV), fracture of the KCl crystal is observed.

When $E_{a}≳E_{c}$, which is the regime relevant to milling, details of collision no longer matter. In this regime, fragmentation is characterized solely by the absorbed energy, as $E_{a}$ is only a small fraction of the initial kinetic energy of the balls (Figure S18). Demonstrating this universality and in addition showing that it extends even to lower energies (below the cohesion energy) is indeed one important result of our simulations in line with previous experimental observation that a specific amount of energy must be absorbed by the sample for reactions to proceed.^[^ ^16^ ^]^ Furthermore, this universality is found to hold also for crystals that are 10 and 70 times larger in volume (Figure S21). This allows us to scale the system and use small balls and a small crystal particle, rather than considering the much larger size of the experimental ball, but also the much larger size of the sample that experiences the collision under experimental ball milling conditions.

Consequently, although the kinetic energies of the milling balls in the simulations are several orders of magnitude lower than those in experiments, they are sufficient to induce complete fragmentation of the crystal particle and disperse the material throughout the nanometric simulation box. Subsequent, typically lower‐energy collisions contribute to mixing of the dispersed material, and the system is thermalized on a timescale of approximately 10 ps (Figure S18). This contrasts with experimental conditions, where, due to the overall mass of the reactants, numerous high‐energy collisions—occurring at a frequency of 30 Hz—may be required to achieve full fragmentation. In experiments, milling balls are also continuously supplied with external energy to maintain both the energy and frequency of impacts. Owing to the larger system size, a greater amount of heat is generated and dissipated, suggesting that mixing is likely driven not only by milling balls, but also by larger reactant fragments. These differences underscore that our simulations are not intended to replicate the entire milling process. Rather, given that the interval between collisions in experiments is on the order of 50 ms, our simulations focus on consequences of a single collision. This enables investigation of the physico‐chemical transformations initiated by mechanical energy transfer, including material fragmentation, scattering, and subsequent reactions, with atomic‐level resolution.

This interpretation is further supported by our analysis of the recombination process. Although the velocity of the fragmented species depends on the energy absorbed during the collision, their mean free path is influenced by the available free volume (99% in simulations vs. 95% in experiments) and the specific geometry of the collision event (see, for example, the insets in Figure 2b). However, due to strong Coulomb interactions, we find that recombination—i.e., the formation of ion pairs and small ion clusters is significant already on the picosecond timescales, and occurs within the cloud of fragmented species generated during the collision. Moreover, the use of periodic boundary conditions in the simulations enables us to resolve the mean free paths of different species and to model the agglomeration and recrystallization processes that occur on longer timescales.

The positive correlation between the imparted mechanical energy and the extent of KCl fragmentation underscores its pivotal role in activating crystalline solids and defines the fragmentation regime in which ball milling operates. According to our simulations, the collision event lasts approximately 10–15 ps, while subsequent reactions including recrystallization within the impacted volume proceed over several tens of nanoseconds (Figure S22). Since this timescale is significantly shorter than the interval between collisions in experiments, recrystallization is likely to compete with complex formation when 18‐crown‐6 ether (18c6) molecules are present.

### Simulations of Dry Complexation: 18c6 with KCl

Motivated by experimental conditions involving the melting of 18c6, we introduce molecules of 18c6 in a 1:1 molar ratio with potassium atoms into the simulation box (Section S2.4 for details on the simulation methodology). The 18c6 molecules spontaneously form liquid droplets, wherein individual molecules commonly adopt the elliptical conformation expected for 18c6 in the condensed state.^[^ ^42^ ^]^

**Figure 3:**
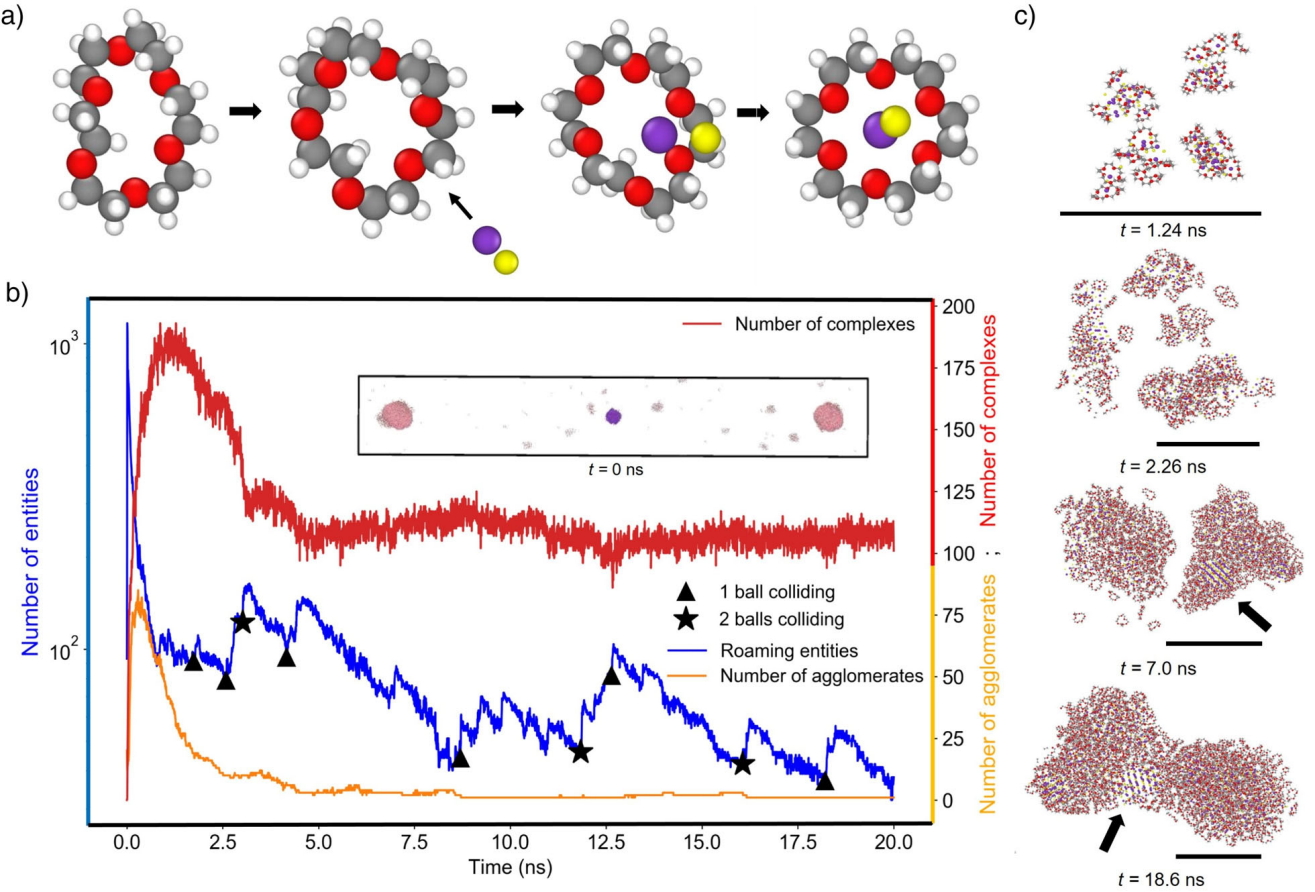
Simulation of the mechanochemical reaction of KCl and 18c6. a) Conformation change in the 18c6 molecule as a KCl ion pair is approaching. b) Time evolution of the number of complexes (molecules of 18c6 containing $K^{+}$ in the cavity) (red), agglomerates (more than five entities that are within a neighborhood of 3.4 Å (orange), and roaming entities (entities that have no neighbors within 5 Å) (blue). Asterisks in the blue curve denote impactful collisions with the aggregates such as 1 ball or 2 balls colliding with an agglomerate. Outcomes of simulations on different KCl crystal particles are shown in (Figure S24). c) Snapshots from simulations showing the coarsening of agglomerates. The arrows point to recrystallized KCl within the agglomerate. The black horizontal bar in each snapshot represents the length of 5 nm.

After the collision and shattering of the KCl crystal particle, the nascent potassium ions and KCl ion pairs interact with the surrounding 18c6 molecules to form complexes (Figure 3a). The initially elliptical conformation of 18c6 opens, orienting the oxygen atoms inward as $K^{+}$ approaches, thereby forming the experimentally observed complex. The $K^{+}$ ion lies slightly above the plane of the oxygen atoms of 18c6 at approximately 0.7 Å, as is commonly observed.^[^ ^32^, ^43^ ^]^ Additionally, the chloride ion attached to the supramolecular complex is found to be inclined relative to the normal to the plane of oxygen atoms, consistent with experimentally determined structure (Figure 1c).

We further analyze product formation over several nanoseconds after the collision by tracking the number of 18c6‐$K^{+}$ complexes (red line and associated axis in Figure 3b). Additionally, we examine the evolution of freely roaming entities (individual crowns and ions) and agglomerates (groups of at least five entities), represented by blue and orange lines, respectively, in Figure 3b (for details of the analysis see Section S2.4.2). We find that the number of complexes increases sharply over a few nanoseconds while the reactants remain highly dispersed in the reaction volume. Due to rapid complexation, the number of freely roaming residues drops within 1 ns, coinciding with the peak in the number of complexes. At this point, the number of agglomerates begins to decrease significantly as a result of the coarsening process. This process is also associated with a decline in the number of complexes, which are destabilized within the agglomerates due to unscreened, competing interactions between potassium and chloride ions. These interactions drive the nucleation of small KCl crystalline domains within the agglomerates (see black arrow in the bottom panel of Figure 3c).

Complex formation halts at approximately one‐third of its maximum possible extent, despite occasional secondary collisions (asterisks and triangles in Figure 3b) that may break large agglomerates into smaller ones, but which leave the recrystallized KCl domains intact. Importantly, the relaxation process is completed within 10 ns, which is orders of magnitude shorter than the 20–30 ms expected between collisions in experiments.^[^ ^44^ ^]^ This clear separation of time scales underscores why the simulated phenomena can be meaningfully compared to experimental conditions. The reaction progresses further if an agglomerate undergoes a new high‐energy collision that shatters it (see Figure S25). This suggests that the mechanochemical reaction advances in tiny discrete batches, each triggered by collisions,^[^ ^9^ ^]^ consistent with the experimentally observed steady formation of the complex under continuous milling (Figure 1a).

### Effect of Water in Wet Complexation of 18c6 with KCl

Finally, we address the observation that the reaction between 18c6 and KCl does not proceed at room temperature in the absence of moisture (Figures 1 and S6), whereas hydrated complexes readily form.^[^ ^32^, ^43^ ^]^ This suggests that water facilitates supramolecular interaction between the crown ether molecules and potassium cations and promotes complex formation. Such a pronounced influence of liquid additives on the kinetics of mechanochemical reactions is commonly reported, sometimes accompanied by changes in selectivity.^[^ ^45^, ^46^, ^47^, ^48^ ^]^ However, the fundamental origin of this effect remains largely unknown.^[^ ^27^ ^]^

**Figure 4:**
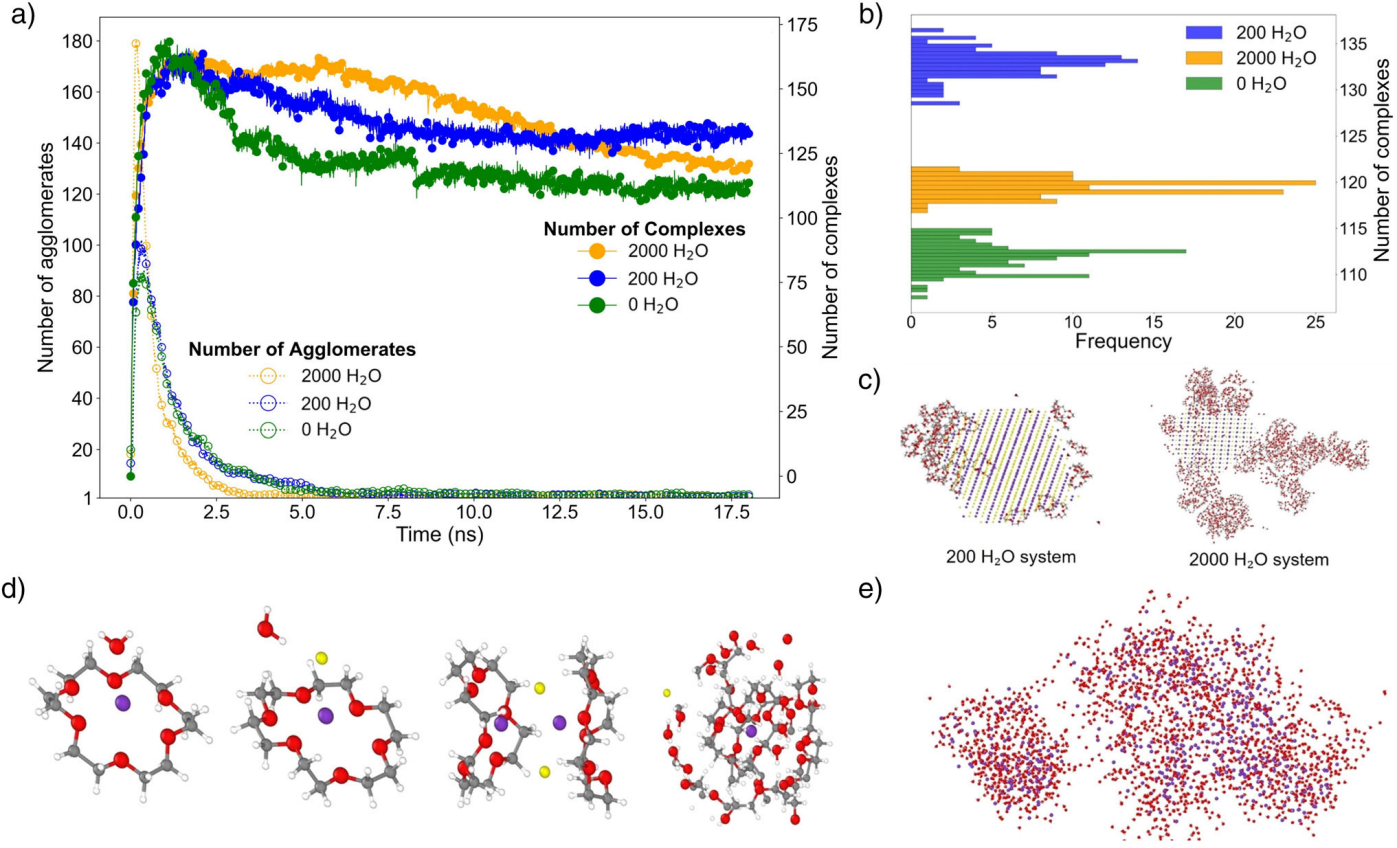
Effect of water on mechanochemical complexation of KCl with 18c6. a) Time evolution of the number of complexes (full circles, axis to the right) and agglomerates (hollow circles, axis to the left) for systems with 2000, 200, and 0 water molecules are shown in orange, blue, and green, respectively. Presented curves are averages over three simulations (for individual runs see Figures S24 and S28). b) Number of formed [18c6${K]}^{+}$ complexes in the steady state, as a function on the water content in the simulations. The most complexes are obtained at the low water content (blue histogram), followed by large water content (orange) and dry conditions (green). c) Adsorption of hydrated 18c6 droplets to KCl crystal corners before the collision. d) Examples of fragments (ensembles of up to four entities within a neighborhood of 3.4 Å) and agglomerates (more than four entities within a neighborhood of 3.4 Å). From left to right: A hydrated [18c6${K]}^{+}$($H_{2}O$) complex, a hydrated complex of 18c6 with a KCl ion pair, a complex dimer, and a complex within an agglomerate. e) Over 90 % of $H_{2}O$ molecules become uniformly distributed in the final agglomerates (only $H_{2}O$ and $K^{+}$ are shown).

We thus, perform simulations where we introduce either a low (200 $H_{2}O$ molecules per 500 KCl ion pairs, i.e., 40 mol%) or a high amount of water (2000 $H_{2}O$ molecules, i.e., 400 mol%) (Section S2.5) and compare wet complexation to the previous dry one. We evaluate the number of complexes as a function of time after collision (full symbols in Figure 4a). Interestingly, high water content facilitates the growth of agglomerates (open symbols in Figure 4a), as evident by the fastest drop in the agglomerate number. More importantly, on time scales of 15 ns after the collision, we find that low‐water content (blue curve) stabilizes the number of product complexes more efficiently, resulting in a higher number of potassium‐18c6 complexes in the steady state, than the high‐water content (orange curve), despite the initially higher number of formed complexes in the high‐water‐content simulation (Figure 4a,b). To rationalize these results, we evaluate the effect of water on both, the reactants and the products.

Both 18c6 and KCl, as well as the complexation product, are hygroscopic solids. Moreover, all crystal structures of the hydrated 18c6 exhibit the open conformation,^[^ ^49^, ^50^ ^]^ indicating that any water present will facilitate opening of the elliptic conformation of 18c6 molecules. Furthermore, prior to the collision, $H_{2}O$ molecules are largely involved in hydrating molecules of 18c6. For example, in the system with high‐water content this is true for 92% of $H_{2}O$ molecules. We also observe adsorption of hydrated 18c6 droplets onto the corners of the KCl crystal particle. This does not occur in the absence of water, and it becomes more prominent with increasing water content (Figure 4c). Still, no spontaneous complexation is observed, consistent with the experimental finding of the absence of complexation in the melt of 18c6 without milling, with both lower and higher water content (see Section S1.3 in the Supporting Information).

Upon a collision, it is instructive to analyze the contents of small fragments (see Section S2.4.2 and Figure S26 for technical details), and hydrated species during coarsening (Figure S27). In the absence of water, the Coulomb interactions remain unscreened, leading to competition between the crown ether molecules and chloride anions for potassium cations. The addition of water introduces new competing effects on the reaction. Namely, water stabilizes both the reactants and the products, to the extent that depends on its concentration. For the low‐water‐content simulations, in the initial 2.5 ns, water molecules help open the conformation of the 18c6 molecules, forming hydrogen bonds toward the 18c6 oxygen atoms. As potassium cations approach, the water molecules are displaced, but remain integrated in the nascent hydrated complex, stabilizing it even in the presence of chloride anions (Figure 4d). As a result, regrowth of KCl is almost completely hindered leaving more $K^{+}$ ions to form complexes in the presence of water (green and blue histograms in Figure 4b).

On the other hand, large amount of water brings in a competition between stabilization of $K^{+}$ as a hydrated species, and $K^{+}$ in a complex with 18c6 (orange histogram in Figure 4b and Section S2.5.2). Consequently, we see the formation of stable species containing hydrated $K^{+}$ at the expense of the number of complexes as the simulation proceeds beyond the initial collision (Figure S27). Thus, too much water, which is uniformly distributed within the agglomerates (Figure 4e), is not as beneficial for the complex stabilization as a small amount of water. This specific case, demonstrating the effect of water as an additive on complex formation, provides a detailed mechanistic explanation for the widely observed but previously unexplained phenomenon in which the impact of a liquid additive peaks at an optimal dosing.^[^ ^51^ ^]^

Although the simulation here reproduce experimental findings, we note that in certain aspects our protocol may depart from a realistic scenario. In particular, one collision may not be representative of the bulk milling process. In the real system additional dissipative paths may take place, which should be studied in the future. Furthermore, the focus in this study was on ionic species, while further effort is necessary to address the challenge of expanding the current protocol to systems that involve the chemistry of the covalent bond.

## Conclusion

In summary, we have developed a simulation protocol that enables a scaled, molecular‐level analysis of physico‐chemical transformation induced by a collision event, typical in ball milling mechanochemistry. We validate our approach through a correspondence with experimental findings and highlight the critical role of energy transfer and crystal fragmentation in generating activated species. The ensuing relaxation process leads to the formation of a complex between $K^{+}$ and 18c6 or the re‐crystallization of KCl. Furthermore, we identify one origin of liquid‐assisted reactivity in ball milling mechanochemistry. A liquid additive can stabilize not only specific products, but also reactants, thereby influencing both the kinetics and thermodynamics of the reaction and suggesting the existence of an optimal liquid additive dosing.

Our findings contribute to a deeper understanding of mechanochemical processes and signify the importance of collisions for reactant activation, but also the immediate surroundings that influence their stability and determine the relaxation pathways. We expect that our protocol will help explore the elusive non‐equilibrium conditions generated by ball impacts and the fundamental mechanisms of mechanochemistry as well as guide the targeted use of liquid additives in mechanochemical reactions.

## Conflict of Interests

The authors declare no conflict of interest.

## Supporting information

Supporting Information

## Data Availability

The data that support the findings of this study are openly available in ZENODO at https://doi.org/10.5281/zenodo.13734954 , reference number 13734954.
