# Supplementary material for: Deciphering Ball Milling Mechanochemistry via Molecular Simulations of Collision‐Driven and Liquid‐Assisted Reactivity
Source: Angew Chem Int Ed Engl. 2025 Oct 23;64(50):e202505263. doi: 10.1002/anie.202505263 (PMC12684300; doi:10.1002/anie.202505263)
Supplement: Supplementary file 1 — Supporting Information [file ANIE-64-e202505263-s001.pdf]

Supporting Information

for

Deciphering Ball Milling Mechanochemistry via Molecular Simulations of Impact-Driven and Liquid-Assisted Reactivity

by

Rupam Gayen[a,†], Leonarda Vugrin,[b,†] Zehua Zhang,[a] György Hantal,[a]
Ivan Halasz,[b] Ana-Sunčana Smith[a,b]

[†] Contributed equally to this work.

[a] PULS Group, Department of Physics, Friedrich Alexander Universität Erlangen-Nürnberg, IZNF, Cauerstrasse 3, 91058 Erlangen, Germany

[b] Division of Physical Chemistry, Ruđer Bošković Institute, Bijenička c. 54, 10163 Zagreb, Croatia

E-mail: ana-suncana.smith@fau.de; ana.smith@irb.hr; ivan.halasz@irb.hr

Contents

S1 Materials and methods **S3**
S1.1 FT-IR spectroscopy S6
S1.2 Room-temperature and elevated-temperature milling experiments S7
S1.3 Control experiments S10
S1.4 Crystal structure solution and validation S13

S2 Molecular modelling **S15**
S2.1 Software and Force fields S15
S2.2 Preparation of the milling balls S15
S2.2.1 Spherical milling balls S16
S2.2.2 Hemispherical milling balls S17
S2.3 Establishing the simulation protocol: KCl fragmentation S18
S2.3.1 Building the KCl crystal particle S18
S2.3.2 Simulation methods S19
S2.3.3 Calculation of absorbed energy by KCl crystal (E_a) S19
S2.3.4 Fragments of KCl S21
S2.3.5 Recrystallization S21
S2.4 Simulations of dry complexation: 18c6 with KCl S26
S2.4.1 Simulation methods S26
S2.4.2 Fragment analysis S27
S2.4.3 Relaxation dynamics S28
S2.5 Wet complexation of 18c6 with KCl S29
S2.5.1 Simulation methods S29
S2.5.2 Fragment Analysis S29
S2.5.3 Relaxation Dynamics S30

S1 Materials and methods

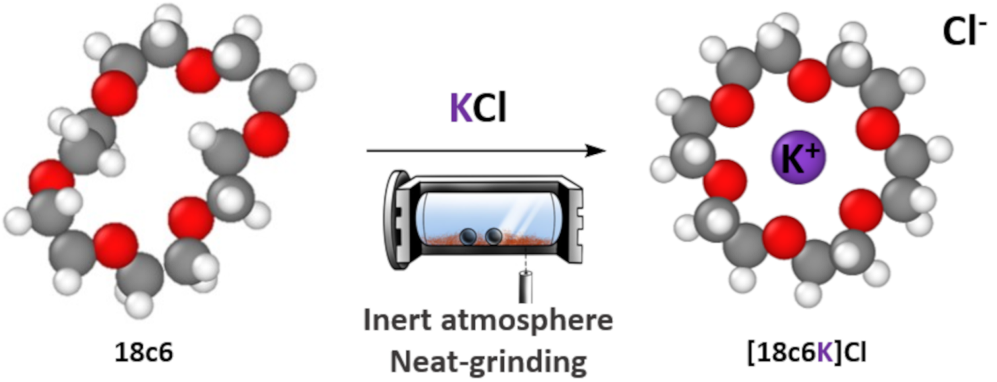

Figure S1: Schematic representation of the complexation reaction between 18c6 (squashed conformation) and KCl. The resulting product is the [18c6K]Cl complex, where the potassium ion is encapsulated by the 18c6 (circular conformation), and the chloride ion remains outside the formed complex.

All materials were purchased from commercial sources and used as received without further purification. The specific chemicals used in this study include 18-crown-6 ether (18c6) (CAS number: 17455-13-9) and potassium chloride (KCl) (CAS number: 7447-40-7), both purchased from Sigma-Aldrich.

Mechanochemical reactions were performed using an IST500 Mixer Mill operating at a milling frequency of 30 Hz. In-house made transparent poly(methyl metacrylate) (PMMA) (internal volume 14.0 mL) milling jars were used along with two stainless steel milling balls (diameter 7.0 mm, weight 1.4 g each). Since the starting materials are hygroscopic, the preparation for chemical synthesis was carried out in a nitrogen-filled glove box ”Vigor Tech USA” on an analytical balance ”Kern”. The conditions inside the glove box were maintained at <0.1 ppm H_2O and <5 ppm O_2. The reaction milling jars were sealed with parafilm and transferred to the vibratory ball mill. Reaction was monitored in situ and in real time using Raman spectroscopy. Upon completion of milling, the reaction jars were transferred to a glove box, opened in an inert atmosphere, and the crude material were collected and prepared for further analysis.

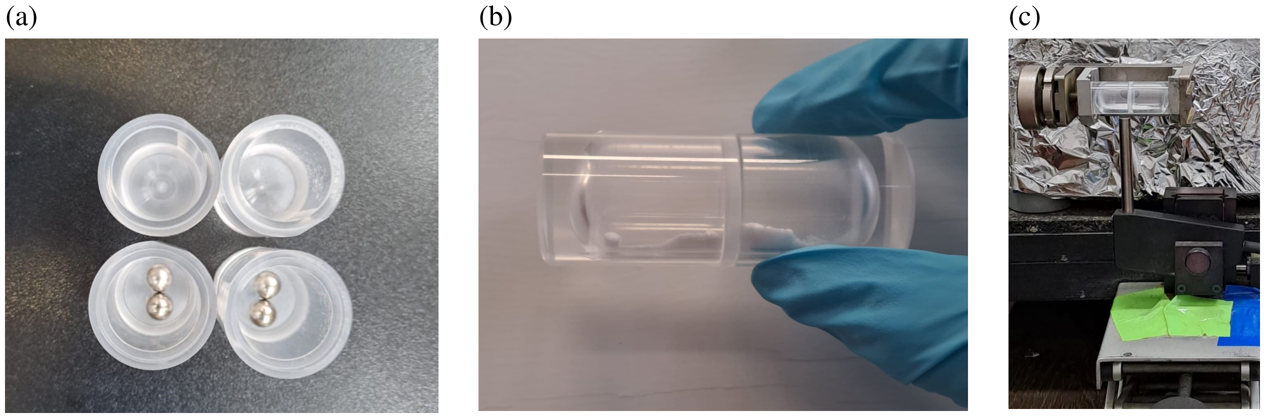

Figure S2: (a) Transparent PMMA jars with stainless-steel (SS) milling balls. b) Reaction PMMA milling jar filled with reaction material. c) Setup for reaction monitoring using Raman probe mounted on the scissor jack below the reaction vessel.

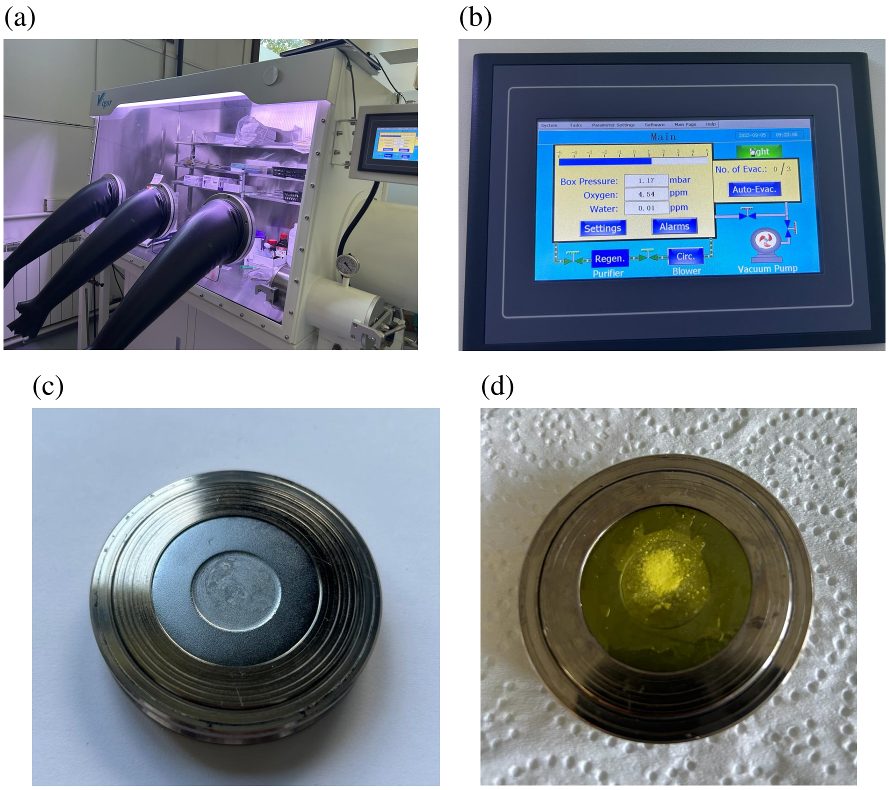

Figure S3: (a) Vigor glove box used for handling air-sensitive materials under a controlled nitrogen atmosphere. (b) Control panel of the glove box showing the monitored parameters, including box pressure, oxygen, and water levels. (c) Empty silicon plate used for PXRD measurements. (d) Reaction product prepared on a silicon plate and covered with Kapton foil to perform the PXRD measurement.

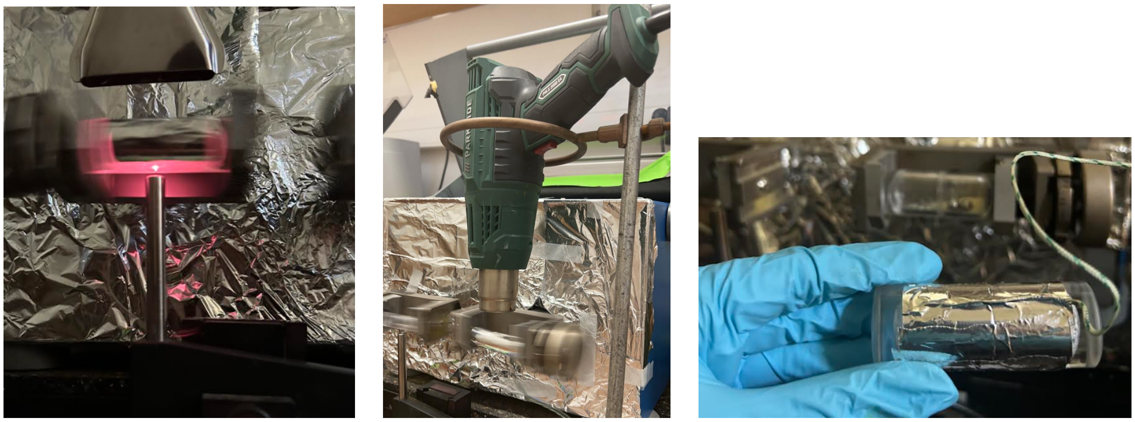

Figure S4: Setup for milling experiments at elevated temperature using a heat gun and a digital multimeter (Metex3660D) for temperature measurements at the outer surface of the milling jar. PMMA milling jar covered with aluminum foil to retain heat, with a thermocouple attached for temperature measurement.

In-situ Raman monitoring was performed using a portable Raman system with a PD-LD BlueBox red laser source with the excitation wavelength of 785 nm, equipped with B&W-Tek fiber optic Raman BAC102 probe, and coupled with an OceanOptics Maya2000Pro spectrometer (with resolution of 1 cm^{-1} or 3.5 cm^{-1}). The probe was positioned about 4 mm below the transparent reaction vessel. The Raman probe was mounted on a stand and equipped with a microscrew for upwards movement that allow precise tuning of the probe's distance from the milling jar. Time-resolved *in-situ* Raman spectra were collected in an automated fashion, with the subtraction of the jar contribution to the Raman spectra done in parallel using an in-house code in MATLAB.[1] Each spectrum was obtained by averaging 8 spectra, each collected suing an integration time of 1000 ms, and the laser power was set up to 485 mW. The laser wavelength was calibrated according to the Raman band of silicon at 520.5 cm^{-1}.

S1.1 FT-IR spectroscopy

Attenuated total reflectance (ATR) Fourier-transformed infrared spectra (FTIR) were collected using a PerkinElmer SpectrumTwo.

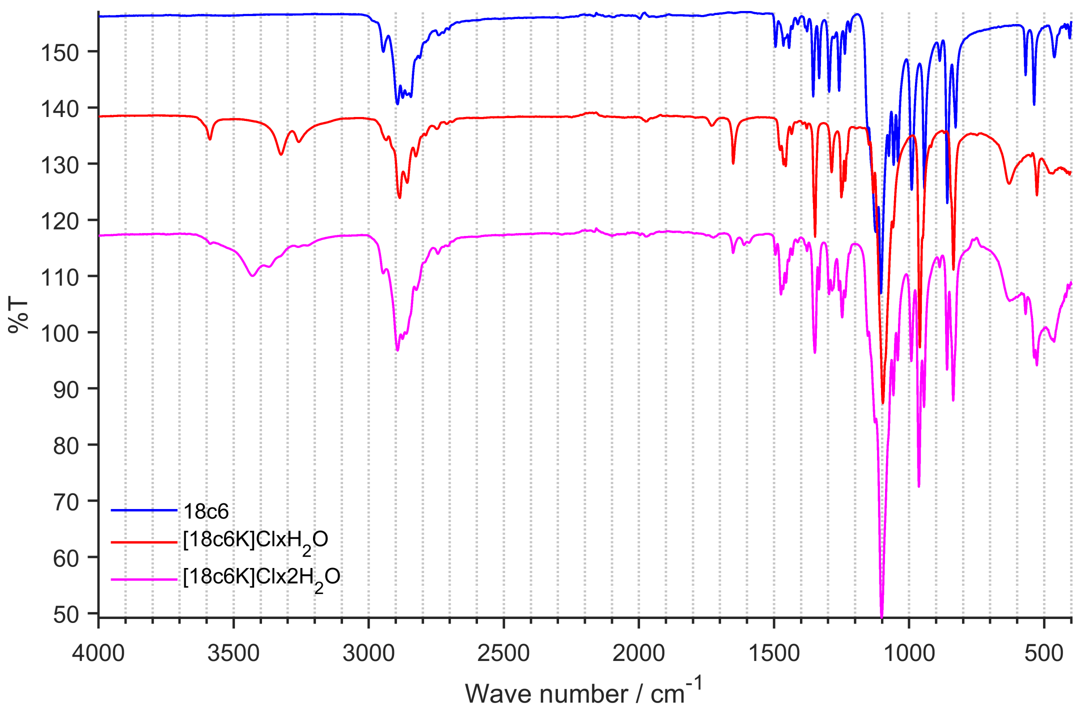

Figure S5: FT-IR spectra comparing (blue line) 18c6, (red line) monohydrate [(18c6)K]Cl complex, (pink line) dihydrate [18c6K]Cl complex. The dihydrate [(18c6)K]Cl complex (pink line) shows a pronounced peak around 3500 cm^{-1}, indicating the presence of additional O-H stretching vibrations due to the extra water molecules.The monohydrate [(18c6)K]Cl complex (red line) also shows a peak in this region, but it is less intense compared to the dihydrate. All three spectra show peaks in the 3000 cm^{-1} region, corresponding to C-H stretching vibrations. The intensity and position of these peaks are relatively consistent across all samples, indicating similar C-H environments. Differences are visible in the 1600-1500 cm^{-1} region, where the dihydrate and monohydrate complexes show additional peaks compared to 18c6. These peaks may be attributed to water bending modes and interactions between the water molecules and the 18c6-K complex. Significant changes can be seen in the 1100-1000 cm^{-1} region corresponding to C-O stretching vibration where the monohydrate and dihydrate complexes show shifts in these peaks, indicating changes in the C-O environment due to complex formation and hydration. The spectra of the monohydrate and dihydrate complexes show increased intensity below 400 cm^{-1}, which can be attributed to lattice vibrations and interactions involving the potassium and chloride ion as well water molecules.

S1.2 Room-temperature and elevated-temperature milling experiments

Room temperature: A mixture of 18-crown-6 ether (18c6) (1.000 mmol, 264.12 mg) and potassium chloride (KCl) (1 equiv., 74.55 mg) was prepared in a nitrogen-filled glove box to prevent moisture absorption. The 18c6 was present in larger crystals which were gently ground using a mortar and pestle. The mixture was then milled in a 14.0 mL PMMA milling jar with two 1.4 g stainless-steel milling balls, each 7 mm in diameter, at 30 Hz. The milling duration was 90 minutes. After milling, the closed reaction container was transferred to the glove box, opened and the solid white material was scraped from the walls of the milling jar and prepared for PXRD analysis (Fig. S8), all in the glove box to prevent exposure to moisture.

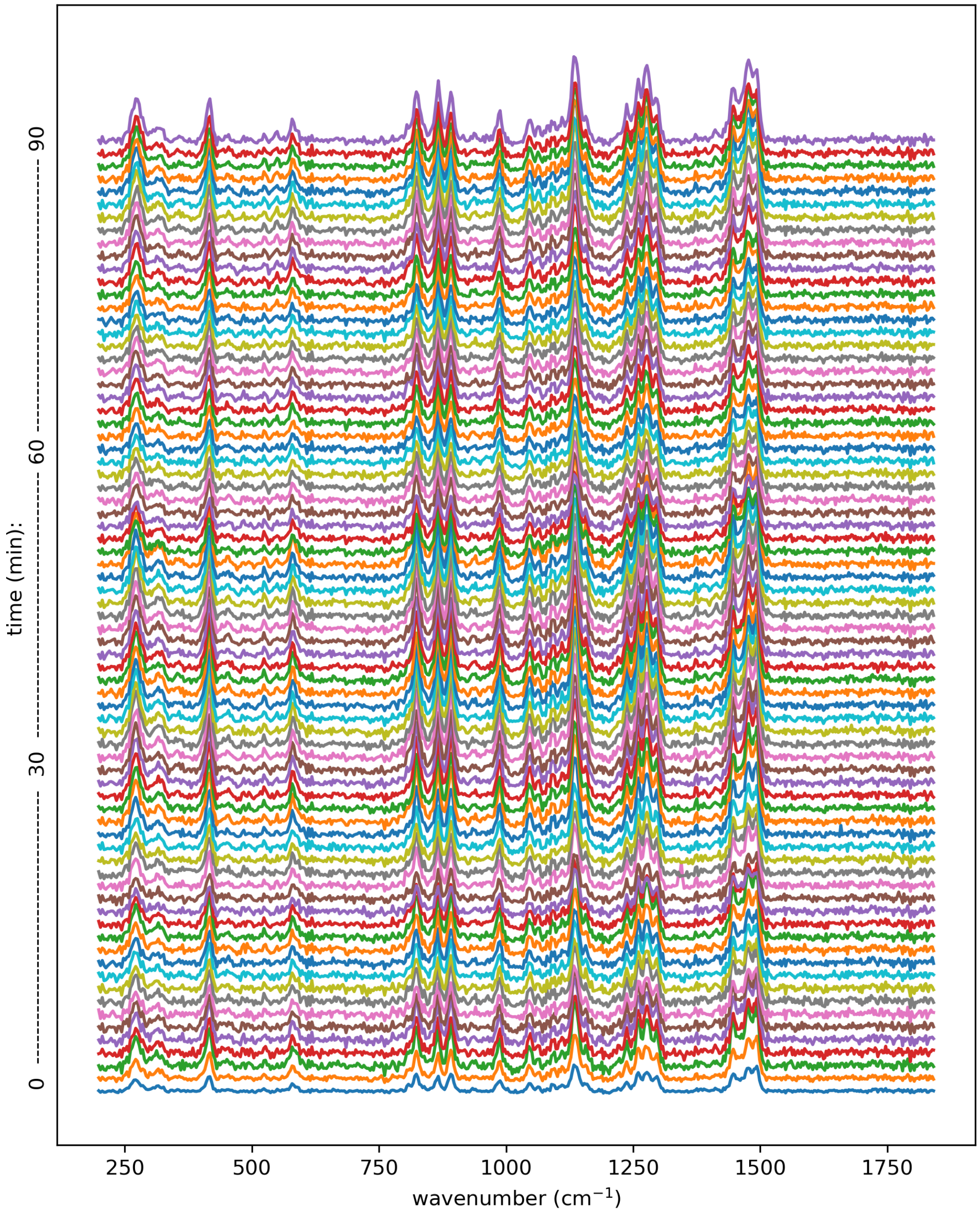

Figure S6: Time-resolved Raman spectra for the mechanochemical neat-grinding reaction of a 1:1 molar reaction mixture of 18c6 ether and KCl at ambient temperature under anhydrous conditions. Milling was carried out at 30 Hz using milling jar made of PMMA (14.0 mL of internal volume) with two SS milling balls (1.4 g) during 90 min. The plot shows the Raman shift over time, indicating there was no complex formation as no significant changes in the Raman spectra were observed during the milling process.

Elevated temperature: A mixture for milling was prepared in the same way as for the experiments performed at ambient temperature. The milling process was carried out with external heating using a heat gun and the temperature of the vessel was maintained at 50°C by adjusting the hot air temperature and the distance of the blower from the reaction container (Figure S3). Since the melting point of 18c6 is ca. 40°C, melting was confirmed both by visual inspection of the transparent vessel and by Raman spectra. The monitoring of milling started before the melting point was reached. After milling, the material was prepared for PXRD analysis as above (Fig. 1b), in the glove box to prevent exposure to moisture.

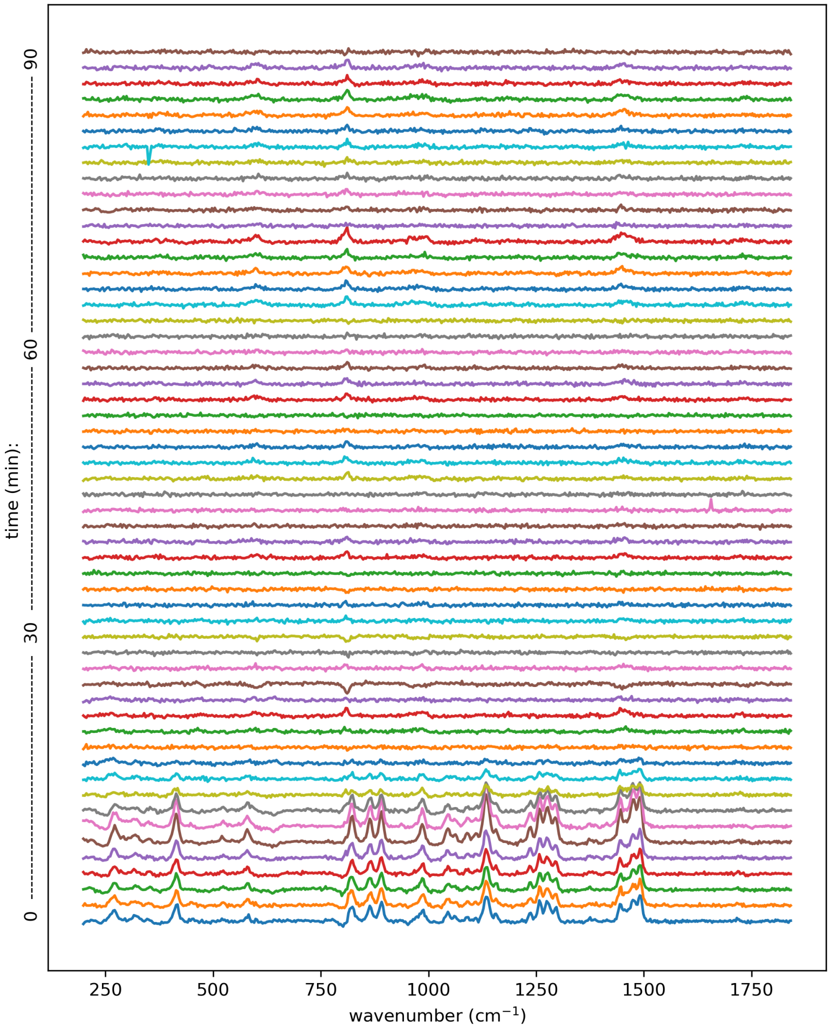

Figure S7: Time-resolved Raman spectra for melting of 18c6 ether under anhydrous conditions, with heating at ca. 50°C with the same milling conditions as for the reaction. Melting occurs after approximately 5 minutes, indicated by a loss of most of the bands of solid 18c6. After melting the initial 18c6 solid was recovered, as analyzed by PXRD, which confirmed that 18c6 experienced only melting.

S1.3 Control experiments

Stirring of KCl in the melt of 18c6 under dry conditions: Finelly dispersed KCl was mixed with 18c6 in thge glove box and was heated until 18c6 melted. The mixture was mixed on a magnetic stirrer for 1 hour after which the mixture was allowed to cool to room temperature.

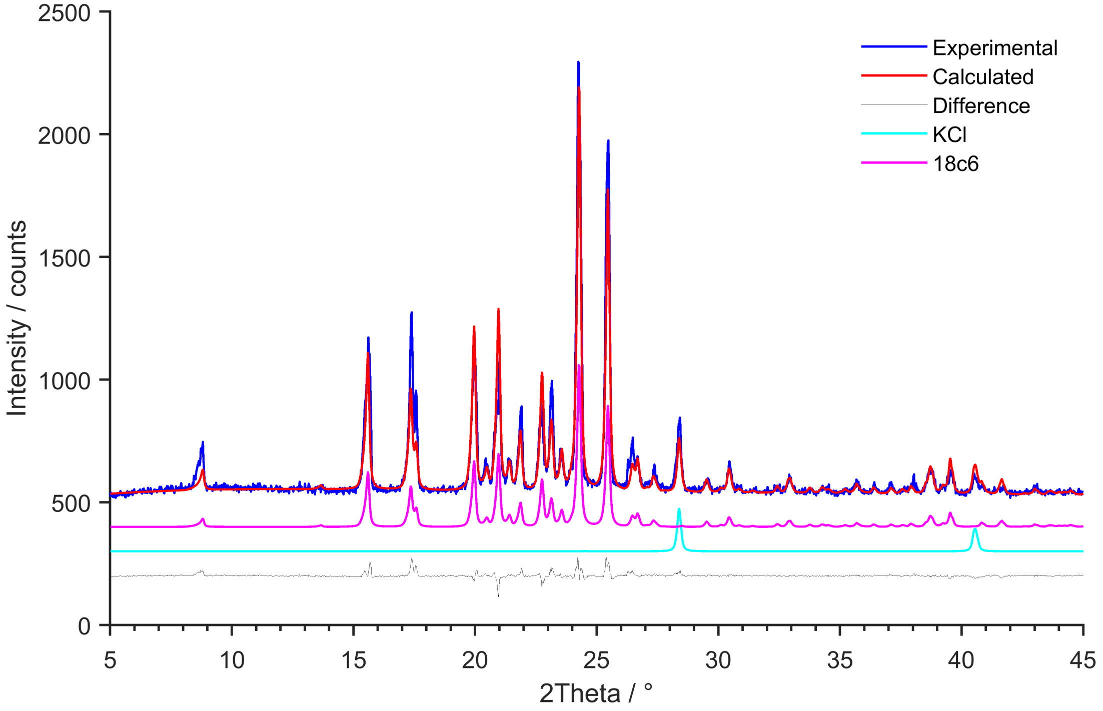

Figure S8: Rietveld fitting of a PXRD pattern collected on a sample that was obtained after stirring of finely dispersed solid KCl in the melt of 18c6. Upon solidification of the melt, the diffractogram consisted of pure phases of KCl (cyan) and 18c6 (magenta) indicating that no reaction occurred.

Stirring of KCl in the melt of 18c6 in the presence of water:

To further investigate potential complex formation under non-mechanochemical conditions, control experiments were conducted by adding precise amounts of water to mixtures of 18c6 and KCl, followed by stirring in the melt of 18c6. All solid materials were weighed under an inert atmosphere inside a glove box and transferred into septum-sealed glass tubes. Distilled water was added by injection through the septum using a microsyringe.

Two different conditions were tested:
a) 40% of water: 18c6 (0.423 mmol, 111.7 mg) and KCl (1 equiv., 31.53 mg) were weighed under nitrogen and transferred into a glass tube. Distilled water (40mol%, 3.1 uL) was added using a 50 uL microsyringe. The mixture was heated above 40 *C* resulting in melting and stirred for 30 min. After stirring, the mixture was solidified by cooling to room temperature and analyzed by PXRD.

b) 400% of water: 18c6 (0.523 mmol, 138.1 mg) and KCl (1 equiv., 38.98 mg) were weighed under

nitrogen and transferred into a glass tube. Distilled water (400mol%, 37.7 µL) was added using a 50 µL microsyringe. The mixture was heated above 40 *C* and stirred for 30 min. After stirring, the mixture was cooled to room temperature and analyzed by PXRD.

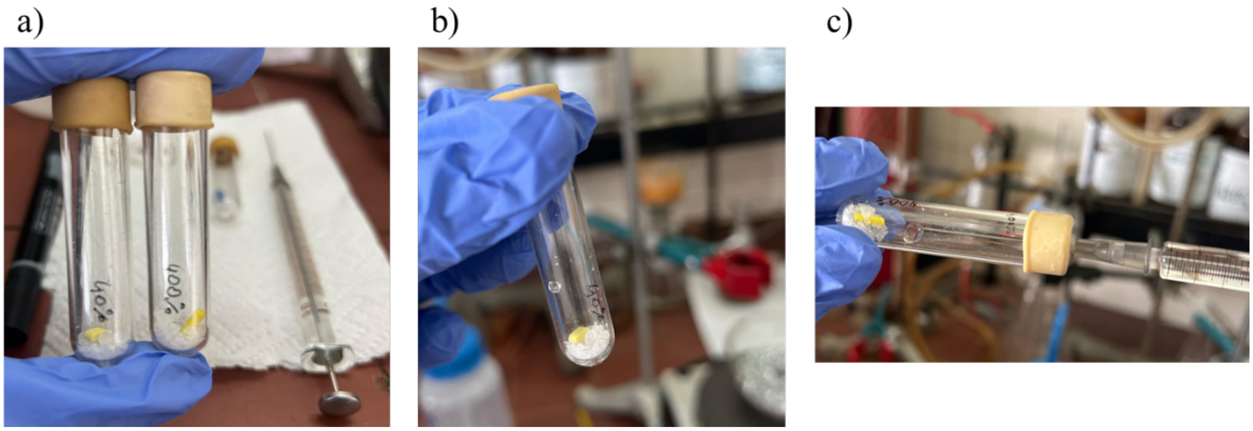

Figure S9: Glass tubes containing 18c6 and KCl prepared for stirring experiments. (a) Mixtures of 18c6 and KCl closed in septum-capped tubes under inert atmosphere before addition of water. b) Addition of 40% water (n = 200 H_2O per 18c6). c) Addition of 400% water (n = 2000 H_2O per 18c6). Yellow bars in the test tubes are magnets that will be used to stir the KCl dispersions in the melt.

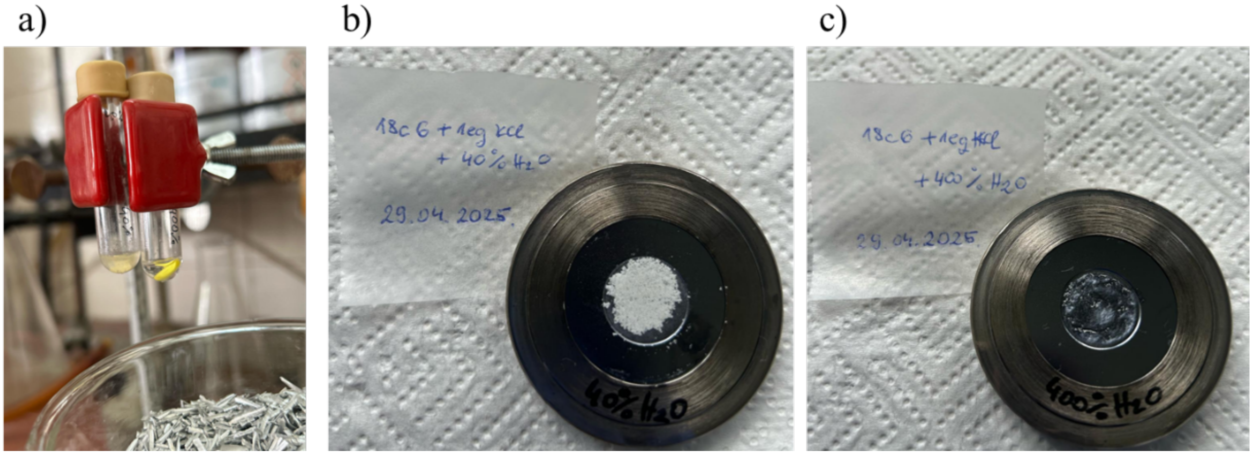

Figure S10: Cooling of reaction mixtures after stirring. The mixture with 40% of water on the left appears as a slurry, while the sample with 400% of water in the right test tube gave a clear liquid. b) Mixture containing 40% water prepared for PXRD, loaded onto a silicon zero-background wafer. c) Mixture containing 400% water mixture prepared for PXRD, also on a silicon wafer. When this sample was allowed to dry in air, a white solid formed.

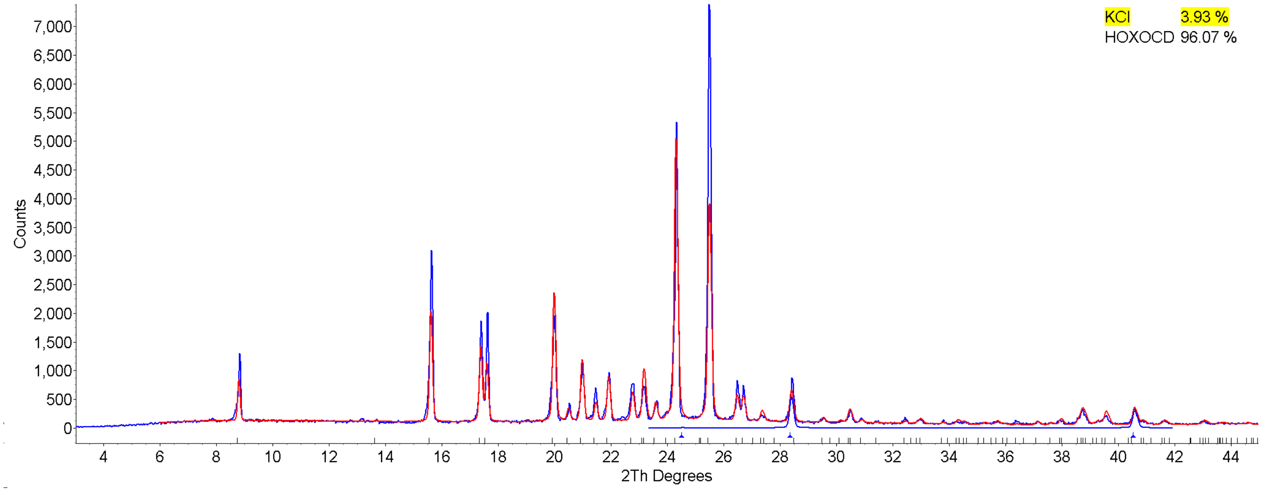

Figure S11: PXRD pattern of the sample obtained by stirring of 18c6 and KCl in the presence of 40 % water per 500 molecules of 18c6) in the melt for 20 min. After cooling to room temperature, the sample was analyzed without further treatment. The experimental diffractogram (blue) is Rietveld-fitted with solid phases of the starting 18c6 (anhydrous, CSD refcode: HOXOCD, dominant contribution) and KCl (its contribution is depicted with the blue calculated diffractogram between the measured pattern and the reflection-position-indicating tick marks). No additional reflections, that could be attributable to any known species of the complex (anhydrous, monohydrate or dihydrate), are present.

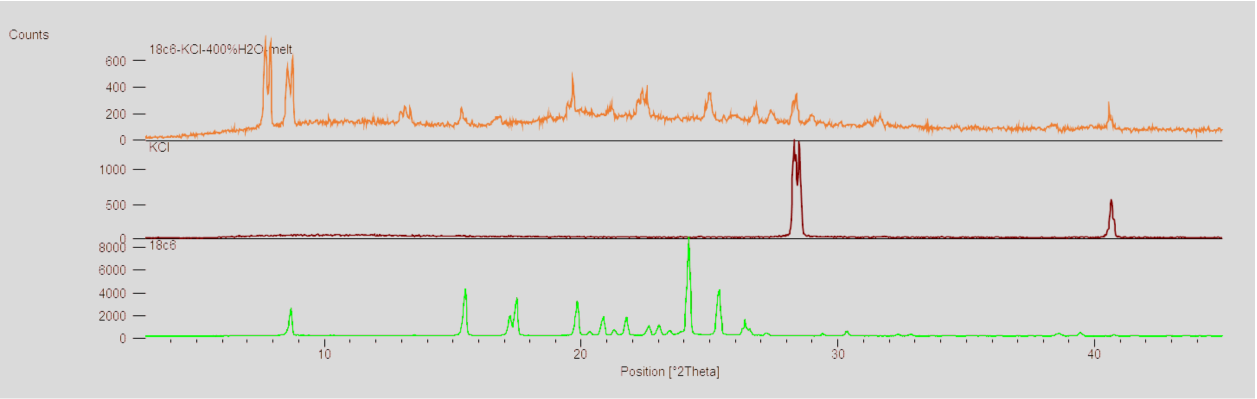

Figure S12: PXRD comparison of the reaction mixture obtained: (orange) still wet sample after stirring 18c6 (264.12 mg, 1 mmol) with KCl (74.55 mg, 1 equiv.) in the presence of 400% water (72.06 µL), (red) KCl starting material, and (green) 18c6 starting material. Reflexes corresponding to KCl are clearly visible (highlighted in grey), while the majority of these signals may originate from some hydrated form of 18c6. Importantly, no evidence of complex formation was detected, as the characteristic signals of the known K^+/18c6 complexes (anhydrous, monohydrate and dihydrate) are absent.

S1.4 Crystal structure solution and validation

Powder X-ray diffraction data (PXRD) for crystal structure solution were collected on a Panalytical Aeris benchtop laboratory powder X-ray diffractometer equipped with a copper X-ray tube operating at 40 kV and 7.5 mA, in Bragg-Brentano geometry, with the sample prepared in a thin layer on a zero-background silicon holder. The sample was covered with Kapton foil to prevent water absorption. Data collection time was set to ca. 1 hour and a repeated PXRD data collection was initiated immediately after the first was finished. This second PXRD pattern exhibited increased peaks from the monohydrate complex $[K_{18}c_6]Cl(H_2O)$ demonstrating the the sample prepared under the Kapton foil, despite having taken due care, absorbed moisture from air. Analysis of the first-collected diffraction pattern indicated that there was the monohydrate present, a small amount of unreacted KCl and an unknown phase.

After accounting for the contributions of the known phases, peaks belonging to the unknown phase were identified and indexed with an orthorhombic unit cell: $a = 11.6233(23)$ Å, $b = 18.1187(41)$Å, $c = 8.2491(17)$Å, $\beta = 90°$, $V = 1737.24(63)$ Å^3. Systematic absences indicated $P2_12_12_1$ space group as a probable candidate. The unit cell volume corresponds well to 4 formula units of the assumed complex ($Z = 4$). Crystal structure solution was achieved using simulated annealing assuming the circular open conformation of the 18c6 molecule and treating as a rigid body, and independent atoms of K and Cl. The crystal structure was recognized when K occupied the cavity of the 18c6 moiety and had a Cl atom attached. Final Rietveld refinements were performed using rigid body description for the 18c6 moiety. All calculations were performed using the program Topas (version 4.2., Bruker-AXS, Karlsruhe, Germany). The cif file for the solved structure was deposited with the Cambridge Crystallographic data center (CCDC) under the number 2343047. These data can be retrieved free of charge from the CCDC upon request. Graphical representations of the structures were prepared using Mercury.[2]

The validity of the crystalline structure obtained from powder diffraction was assessed by periodic DFT calculations using the freely available SIESTA 5.0 software (Figures S13 and S14).[3, 4] The calculations were performed using localized atomic orbitals constructed with double zeta basis functions augmented with a polarization function added for each element. The PBE exchange-correlation functional [5] was used in conjunction with Grimme's dispersion corrections.[6]

Initial calculations were set up with standard pseudopotentials retrieved from the SIESTA pseudo potential database.[7] Due to the low quality of the K pseudo potential flagged by SIESTA, new pseudo potentials were generated for K, by readjusting the pseudo core radius of the 4s orbital using the ATOM software.[8] The generated K pseudo potentials were bench marked by checking the lattice constant of the optimized KCl structures against the experimental value. The transferability of the finally chosen K pseudo potential was assured by comparing the predicted energies of various electron configurations with those determined in all-electron calculations. Subsequently, conjugate gradient geometry optimization was performed using periodic boundary conditions in all 3 directions with convergence criteria of 10^{-3} eV, 10^{-2} eV/Å, 10^{-4} and 0.1 GPa applied in the energy, the forces, the density matrix elements and the stress components, respectively. The stability of the optimized structure was assessed by assuring that the computed phonon spectrum had only real frequencies.

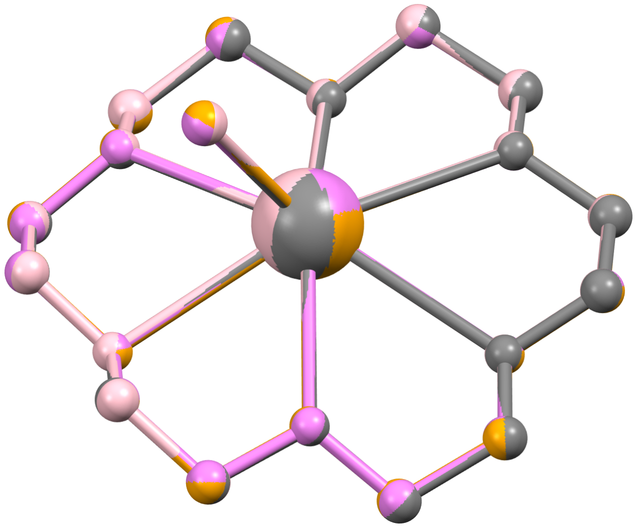

Figure S13: Overlay of 4 molecules of the [18c6K]Cl complex in the unit cell as obtained after DFT optimisation in the space group $P1$. Close overlap confirms the correctness of the space group $P2_12_12_1$ in which the crystal structure was solved.

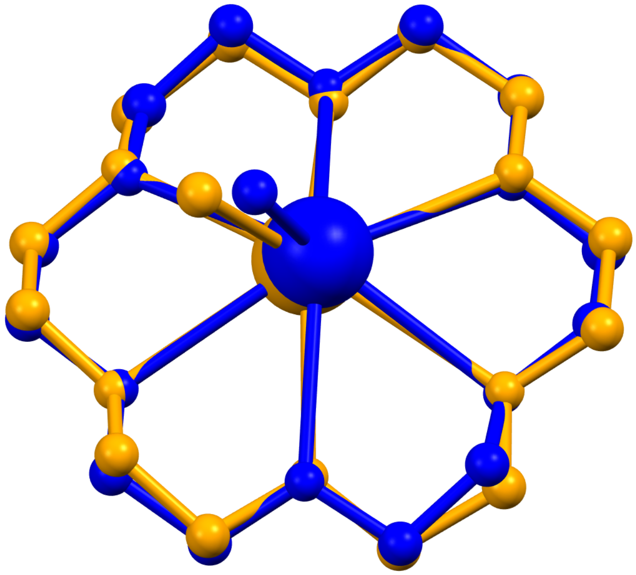

Figure S14: Overlay of DFT optimised molecule (yellow) and the experimental (blue) conformation refined against PXRD data. The close overlap served to validate the experimentally determined molecular conformation and the crystal structure.

S2 Molecular modelling

S2.1 Software and Force fields

The dynamics of the mechanical processes were studied using GROMACS 2021.5 with the OPLS-AA force field for all the species. [9, 10, 11, 12, 13] Simulations employed the leap-frog algorithm for integrating Newton's equations and a velocity-rescale thermostat for thermalization. During milling, the system's initial high energy is dissipated by coupling it to a thermostat, which controls all species except the milling balls. In this way, the balls get thermalized only indirectly, through the other species. The 18C6 ether was parameterized using OPLS-AA parameters via the LigParGen server using localized bond-charge corrected CM1A charges (1.14*CM1A-LBCC) which can be found in folder 18C6 parameters in Zenodo - 10.5281 zenodo.13734954.[14, 15, 16]. LigParGen automates OPLS-AA parameter generation for small molecules using CM1A, 1.14CM1A, and CM1A-LBCC charge models. It supports input formats like SMILES, PDB, MOL, and MOL2, and provides topology and coordinate files compatible with Gromacs and other software. Liquid water was modeled with the extended simple point charge (SPC/E) model, which provides accurate structural and thermodynamic properties [17] The simulations were visualized and captured as images using Open Visualization Tool (OVITO) and Visual Molecular Dynamics (VMD). [18, 19, 20]. All input files and parameters are available on Zenodo - 10.5281/zenodo.13734954.

Non-bonded interactions were modeled with a 12-6 Lennard-Jones potential and the parameters are tabulated in Table-S1. [9, 10] All input files are available in the Section: Building Milling ball of folder "Ball Milling Paper" in Zenodo - 10.5281/zenodo.13734954.

Table S1: Non-bonded parameters

**Ion**	Mass	σ(nm)	ε(kJ/mol)	z(e)
**Milling Ball**				
Atom 1	85.4678	0.562177	0.000715464	0
Atom 2	79.9040	0.462376	0.376560	0
**KCl Crystal**				
K+	39.09830	0.493463	0.00137235	+1
Cl-	35.45300	0.441724	0.492833	-1

S2.2 Preparation of the milling balls

Milling balls play an important role in mechanochemical reactions, yet the size and velocities of the balls used in the experiments make their explicit modeling impossible in molecular dynamics. We, therefore, aim at creating objects that fulfill the purpose of experimental milling balls and make an efficient transfer of kinetic energy.

Several design principles were employed:

1) Because the milling balls' material should not contribute to the chemical reaction, the chemical composition of the balls is not considered relevant, and a model material has been created.

2) The stiffness and toughness of the structure should be such that no fragmentation or major plastic shape deformation are observed. Likewise, bulk modulus that determines the extent of the elastic deformation of the ball during the collision, should be large to secure efficient energy transfer.

3) No particular symmetry axis should exist in the ball. If such an axis exists, its orientation relative to the crystal axis of the reactant at the collision could affect the energy transfer and the reproducibility of the simulation.

4) Random chemical roughness is allowed, but no detachment of individual atoms. This is motivated by the fact that in experiments, the materials of the balls is selected for hardness and wear resistance. Although high-energy collisions may cause minor atomic peeling, the ball material is chosen such to prevent these fragments from influencing the reaction.

5) The balls should not be a source of chemical potentials but rather behave as hard spheres.

To design balls for our *in silico* model that satisfies all these requirements, we also restricted ourselves to tools easily accessible to MD simulation packages such as GROMACS. We note that the aim here was not to create a ball that has a resemblance to a true material. Our goal is to create designs that emulate the role of balls in a milling device and achieve very high kinetic energies (compared to kBT), while maintaining structural integrity – given that velocity range is limited by the integration time scales of femtoseconds (relevant for modeling molecular complexation), and that size of the milling ball is restricted to the nanoscale, designs were associated with artificially high densities.

The topologies, and force field parameters are provided on Section: Building Milling ball of folder "Ball Milling Paper" in Zenodo - 10.5281/zenodo.13734954..

S2.2.1 Spherical milling balls

For the preparation of spherical milling balls a total of 17,972 pairs of oppositely charged monoatomic ions (molecular mass: 165.37 g/mol per pair) were introduced into a simulation box. Annealing was performed from 273 K to 2600 K over 12 ns. The temperature was maintained at 2600 K for 4 ns, then slowly decreased to 2000 K within 14 ns, resulting in an amorphous elliptical structure. Upon visual inspection, we carved out a spherical shape composed of 10732 ion pairs, while the remaining ions were removed using VESTA. [21] In the next step, energy minimization was performed on this spherical structure using the steepest descent algorithm keeping the electrostatic potential between the ions intact. This step relaxes the shape of the interface. In the next step, charges of all the ions of the minimized structure were assigned zero charge such that they become neutral.

The minimized structure was then constrained by creating harmonic bonds of 3 Å between any particles within 7 Å of each other. These bonds were assigned an exceptionally high force constant of 600,000 kJ/(mol·nm^2) to ensure the rigidity of the resulting structure. The modified bonding information was incorporated into the topology file, and a subsequent round of energy minimization was performed to relax the system under these new constraints.

During this second energy minimization, the strong harmonic potentials induced significant struc-

tural rearrangements due to the competing demands of multiple strong restraints acting simultaneously, forcing atoms into closer proximity and a number density of 123.4 atoms/nm^3. The result was a significant volume constriction and a tightly interconnected network of particles, where the mechanical stability is predominantly governed by the harmonic potentials of the artificial bonds. Consequently, the system formed a neutral, rigidly constrained amorphous dense ball with an approximate radius of 35 Å and a mass $3.5 * 10^6$ amu as shown in Figure S15 (left). While among each other, atoms constituting the ball interact only by harmonic springs, interactions with the environment are captures by van der Waals interactions (as shown in SI Table 1) . The ball was duplicated so that each milling simulation included two balls.

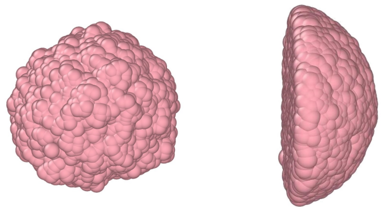

Figure S15: Milling ball designs used in simulations. All designs are neutral, rigidly constrained structures of (left) spherical or (right) hemispherical shape.

S2.2.2 Hemispherical milling balls

To investigate the role of the curvature at impact we created hemispheres to represent milling balls. For their preparation, four of the existing balls were positioned close to each other in a simulation box and subjected to simulated annealing, quadrupoling the initial volume. The procedure mentioned above was repeated to obtain a neutral, rigidly constrained amorphous spherical ball. A segment of this already constrained sphere was extracted, consisting of 21464 neutral atoms using python scripting. Hence the mass of the hemispherical design matched that of a sine spherical ball as shown in Figure S15 (left).

To achieve even greater structural rigidity and prevent unwanted large deformations, we reintroduced the constraints in the hemisphere. Specifically, another set of harmonic springs with the rest length of 3 Å and a force constant of 600,000 kJ/(mol nm^2) was imposed between any particles at the distance of 7 Å. A third round of energy minimization was performed upon incorporating the bonding information in the topology file. The constraints yield further constriction of the volume (481.5 atoms/nm^3), and an even stiffer design.

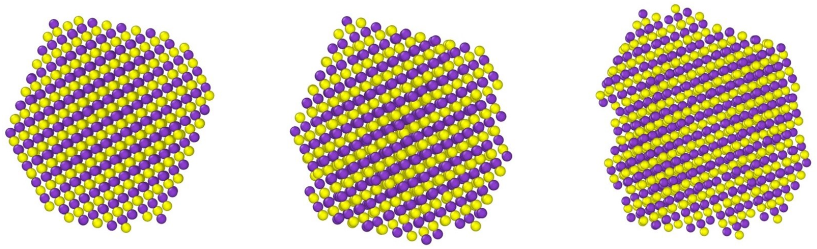

Figure S16: Three distinct KCl crystal particles with 500 K^+ and 500 Cl^- ions used as starting configurations.

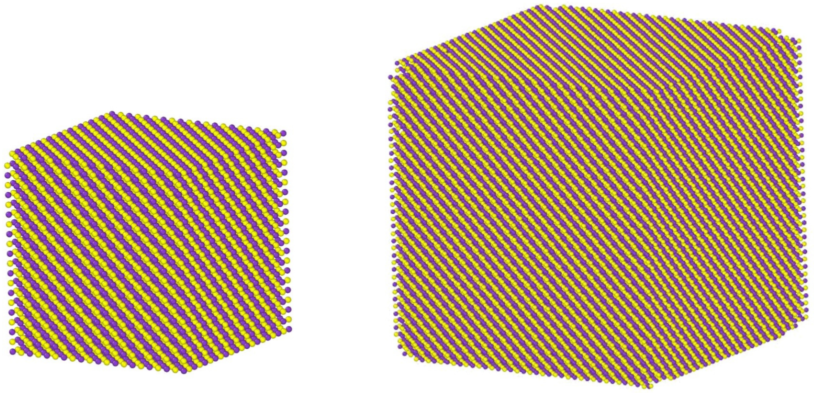

Figure S17: Larger KCl crystal particles used in scaling of fragmentation of KCl with (left) 4360 ion pairs and (right) 34419 ion pairs. Relavant for Figure S21 below.

S2.3 Establishing the simulation protocol: KCl fragmentation

S2.3.1 Building the KCl crystal particle

To model K^+ and Cl^- ions, we use the OPLS force field, with the Lennard-Jones parameters of the ions are tabulated in Table-S1. [9, 10]. The KCl crystals were prepared by placing 500 K^+ and Cl^- ions at random positions within a cubic box with dimensions of ($x \times y \times z = 10 \times 10 \times 10$ nm^3) using gmx-insert molecules tool.

In the following, energy minimization was performed using the steepest descent algorithm which resulted in poorly structured linear chains of KCl ion pairs. Further equilibration involved an NVT MD run at 273 K for 5 ns with the 1 fs time step. The time constant for the thermostat was set to 2ps. This step resulted in a poorly crystalline structure. To rectify this, simulated annealing was performed for 30 ns, increasing the temperature from 273 K to 1800 K over 12 ns, maintaining 1800 K for 3 ns, and then decreasing slowly to 273 K over 15 ns. This yielded a face-centered cubic KCl crystal with a 6.3 Å lattice constant, consistent with experimental values. The procedure was repeated thrice to generate three unique crystal configurations presented in Figure S16.

The face-centered cubic KCl crystal obtained above was further utilized to construct two larger crystals. This was achieved by periodic translation of its unit cell while preserving the lattice constant along the x, y, and z directions using VESTA. Thus two KCl supercells were generated, and subsequent energy minimization yielded face-centered cubic KCl crystals containing 9,260 and

68,838 atoms, respectively (Figure S17).

All input files are available in Section : Building KCl crystal of folder "Ball Milling Paper" in Zenodo - 10.5281/zenodo.13734954.

S2.3.2 Simulation methods

To simulate the milling of KCl crystal, two balls and the previously prepared 1,000-atom crystals were placed in a rectangular prism-shaped box ($130 \times 20 \times 20$ nm^3). The 9,260-atom crystal was assigned to a slightly larger box of dimensions $150 \times 30 \times 30$ nm^3, while the 68,838-atom crystal was placed in a $150 \times 50 \times 50$ nm^3 box. In all cases, the crystal was centered within the box, and the milling balls were positioned on diametrically opposite to the crystal at a distance of 55 nm for the smaller crystals (1000 atoms) and 65 nm for the larger crystals (9260 and 68838 atoms). All configurations were generated using the gmx editconf tool. For equilibration, an MD run in the NVT ensemble was performed for 5 ns with the balls frozen at their position, while the KCl particle was thermalized at 273 K while removing its center of mass translational velocity, exciting vibrational phonon modes of the crystal. To efficiently generate the appropriate velocity distribution, 0.1 ps coupling constant was chosen for the thermostat.

For the milling run, equal and opposite initial velocities from 0.1 nm/ps to 10 were assigned in the gro file for all atoms in the milling balls to ensure collision with the KCl crystal. For the largest crystal (68,838 atoms), higher impact velocities ranging from 1 nm/ps to 24 were applied to the milling balls, under the assumption that higher energies would be required to drive the system into fragmentation regime. The milling runs were performed at 273 K for 1 ns with a time step of 0.1 fs and time constant of 2 ps for the velocity-rescale thermostat. This longer coupling constant allows to allow more natural fluctuations.

All input files are available in Section: Milling only KCl crystal of folder "Ball Milling Paper" in Zenodo - 10.5281/zenodo.13734954.

S2.3.3 Calculation of absorbed energy by KCl crystal (E_a)

Energy absorbed by the KCl crystal $E_a(total)$ during impact determines its mechanical response. It is quantified as the difference between the maximum total energy of the ions building the crystal observed during the collision $E_{\text{kin+pot}}(collision)$ and the total energy of the crystal before the collision $E_{\text{kin+pot}}(crystal)$

$$E_a^{\text{total}} = E_{\text{kin+pot}}(collision) - E_{\text{kin+pot}}(crystal). \tag{1}$$

The time evolution of the total energy of the ensemble of K^+ and Cl^- ions $E_{\text{kin+pot}}(t)$ was obtained as the sum of the instantaneous kinetic $E_{\text{kin}}(t)$ and potential energy $E_{\text{pot}}(t)$. The kinetic energy $E_{\text{kin}}(t)$ of the ions throughout the simulation using the MDAnalysis suite in Python[22, 23] as

$$E_{\text{kin}}(t) = \sum \frac{1}{2} m v(t)^2, \tag{2}$$

where m and $v(t)$ corresponds to the mass and the instantaneous speed of the ion at the time t. The summation is performed over all (K^+ or Cl^-) in the system.

The potential energy $E_{\text{pot}}(t)$ of the ions was determined using the gmx energy tool in GROMACS. [24] by summing the contribution from dispersive interactions $V_{\text{LJ}}(t)$ and the Coulomb interaction $V_{\text{c}}(r)$:

$$E_{\text{pot}}(t) = V_{LJ}(t) + V_c(t). \tag{3}$$

The dispersion interaction is estimated by the Lenard Jones interaction energy

$$V_{LJ}(t) = \sum_{i>j} 4\varepsilon_{ij} \left[\left(\frac{\sigma_{ij}}{r_{ij}(t)} \right)^{12} - \left(\frac{\sigma_{ij}}{r_{ij}(t)} \right)^{6} \right]. \tag{4}$$

Here, $r_{\text{ij}}(t)$ represents the instantaneous distance between two ions i and j, ε_{ij} is the depth of the potential well and σ_{ij} is the finite distance at which the potential between ions V_{LJ} is equal to zero.[25] In our system, for the interaction of ions of the same type, ε and σ are given by the OPLS force field, while for the interaction of K^+ and Cl^-, we use the geometric average of parameters characterizing interactions of K^+ with K^+ and Cl^- with Cl^-, as appropriate for the OPLS force field.

Coulomb interaction is calculated as

$$V_c(t) = \sum_{i>j} k \frac{q_i q_j}{r_{ij}^2(t)}. \tag{5}$$

Here, the electric constant is denoted by k and q_i and q_j are charges of the ion (+1 for K and -1 for Cl).[25]

To extract E_a^{total} from simulations, we determine the time t of the collision as the time in which $E_{\text{kin+pot}}(t)$ experiences a sharp maximum (orange dashed line in the inset of S18) yielding $E_{\text{kin+pot}}(collision)$, while $E_{\text{kin+pot}}(crystal)$ is evaluated just before the onset of the collision (green dashed line in the inset of S18).

Finally, we normalize the E_a^{total} by the number of ion pairs in the system $n_{\text{ion pair}}$ to obtain $e_a^{\text{ion pair}}$:

$$e_a^{\text{ion pair}} = \frac{E_a^{\text{total}}}{n_{\text{ion pair}}} = \frac{1}{500}\left(E_{\text{kin+pot}}(collision) - E_{\text{kin+pot}}(crystal)\right). \tag{6}$$

$e_a^{\text{ion pair}}$ can be directly compared to the cohesion energy of the crystal as shown in Figure 2 of the manuscript.

We note here that the total energy of the ions is a small fraction of the total energy of the entire system, which includes the contribution from the milling balls. In the collision, the milling balls loose some of their kinetic energy to the reactants but also internal deformations, but the total energy of the system (balls +reactants) is strictly preserved throughout the run.

A specific example of this energetic calculation of KCl crystal, when spherical milling balls are moving at an initial velocity of 10 nm/ps is presented in Fig. S18. This analysis was carried out for all initial velocities of the milling balls to investigate the dependence of fragmentation of the KCl crystal on the energy absorption (Figure 2 in the manuscript).

S2.3.4 Fragments of KCl

In the milling simulations of KCl crystal and balls, the KCl crystal is broken down into fragments depending on the absorbed energy by KCl crystal (detailed in Section S2.4). Here we define fragments as individual ions or a collection of ions which have a neighbor within 3.9 Å. This distance criterion of 3.9 Å is chosen such that it exceeds the lattice constant of the distance between K+ and Cl- (3.15 Å), while still still being just below the distance of the second neighbor in the KCl crystal (4.1 Å). This allows us to capture somewhat disordered structures, without double-counting neighbors.

To further understand the fragmentation process, we investigated how the curvature of milling balls influences impact dynamics. Using the hemispherical milling balls we prepared, we assessed the mechanical response of the KCl crystal by colliding the balls on both their curved and flat sides (see green and blue points in Fig. S19, respectively), while keeping all other parameters constant. We then evaluate the number of fragments as a function of kinetic energies of the milling balls (Fig.S19).

At low kinetic energies, planar geometry proves more efficient in fragmenting the crystal compared to curved geometry — a result that may initially appear counterintuitive. This enhanced efficiency is likely due to the more uniform distribution of momentum across the entire crystal particle. The reduction in the escape volume results in higher kinetic energies for the fragments ejected from the contact region. Additionally, the velocity dispersion (in both magnitude and direction) is significantly smaller than that observed after the impact of a curved surface. However, at high impact velocities, these distinctions become negligible, as both collision geometries lead to complete fragmentation of the crystal.

The extent of fragmentation is not only influenced by the collision geometry but also by the initial kinetic energy of the milling balls. Regardless of ball design, three distinct impact outcomes were observed : at the lowest kinetic energies and small momenta of milling balls, the crystal, upon collision, exhibits vibrations without breaking (see top row of Figure S20). At slightly higher kinetic energies and momenta, the crystal fractures into 2-5 fragments (see second row of Figure S20). At larger momenta, the crystal undergoes fragmentation (see third row of Figure S20).

S2.3.5 Recrystallization

Following a high-energy impact, we observe the recrystalisation of the KCl particles on time scales of about 10 ns. The speed of the coarsening process is captured by the decaying number of fragments shown by the blue line in Figure S22 for a systems with milling balls initially set at 10 nm/ps. In the early stages of recrystalisation K and Cl ions form poorly structured fragments (see snapshopt at 1 ns post collision in S22). As the fragments become bigger (snapshots at 3 and 6 ns), they start to show local FCC symmetry but also a lot of defects. As the coarsening progresses, the secondary collisions aid to remove these defects and produce larger domains with FCC symmetry (10ns), a process that will continue until only 1 crystal particle is present in the system.

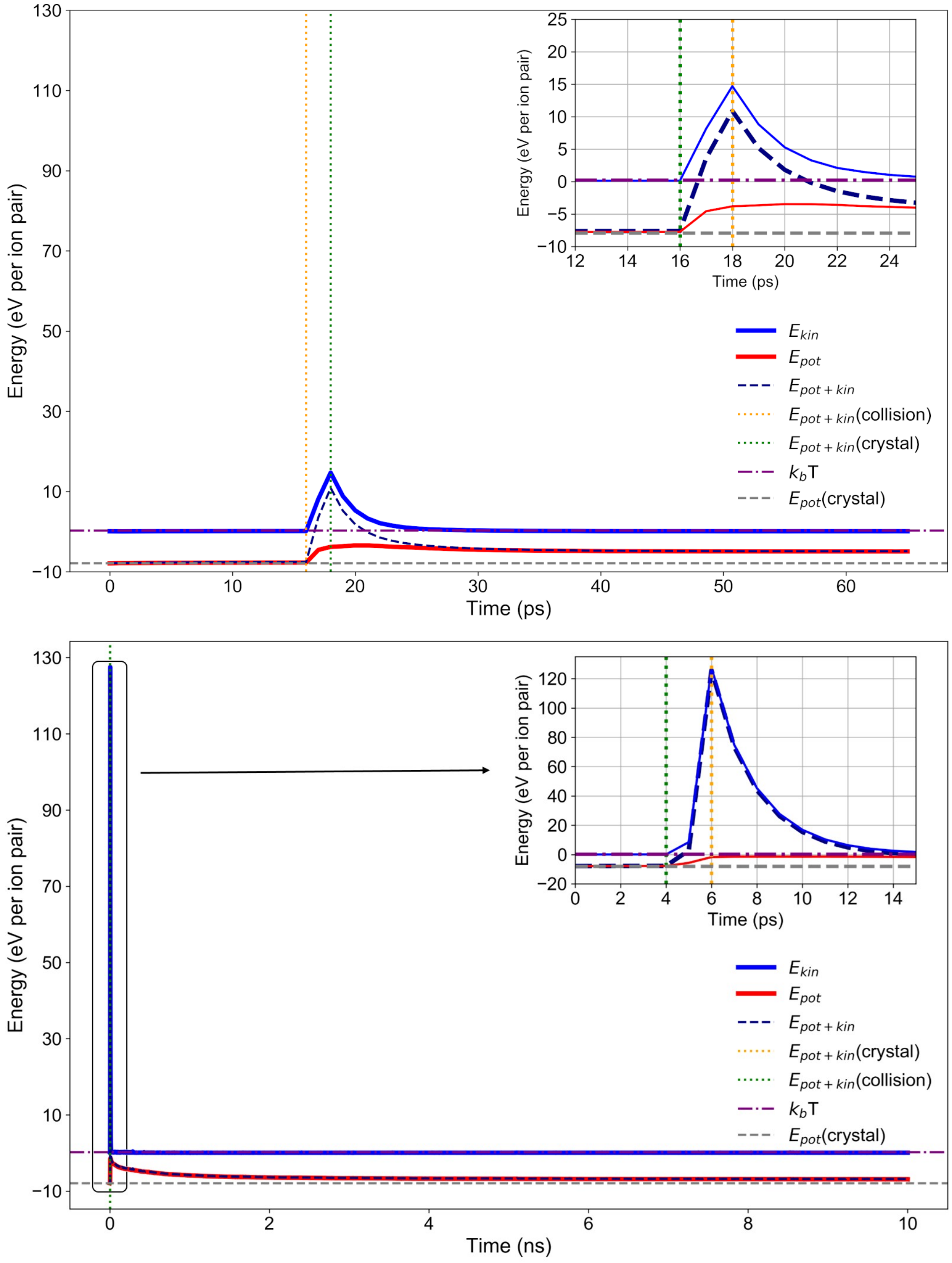

Figure S18: Energy components of all K^+ and Cl^- ions as a function of time during a mechanochemical collision induced by two model milling balls moving at equal and opposite velocities of (top) 3 nm/ps and (bottom) 10 nm/ps. The inset plots magnify the change of the energy terms during the collisions (for slower balls the collision lasts longer). The sharp rise in the total kinetic energy upon impact along with the increase of potential energy as the crystal breaks during the collision. The difference between the total energy after the collision (orange dashed line) and the total energy before the collision (green dashed line) provides the amount of energy absorbed by the KCl crystal during the impact. The slower impact velocity (top graph) provides energy absorbed per ion pair at around the cohesion energy of KCl crystal and the higher impact velocity (bottom graph) is well above the cohesion energy and in the regime of full fragmentation of the KCl crystal particle.

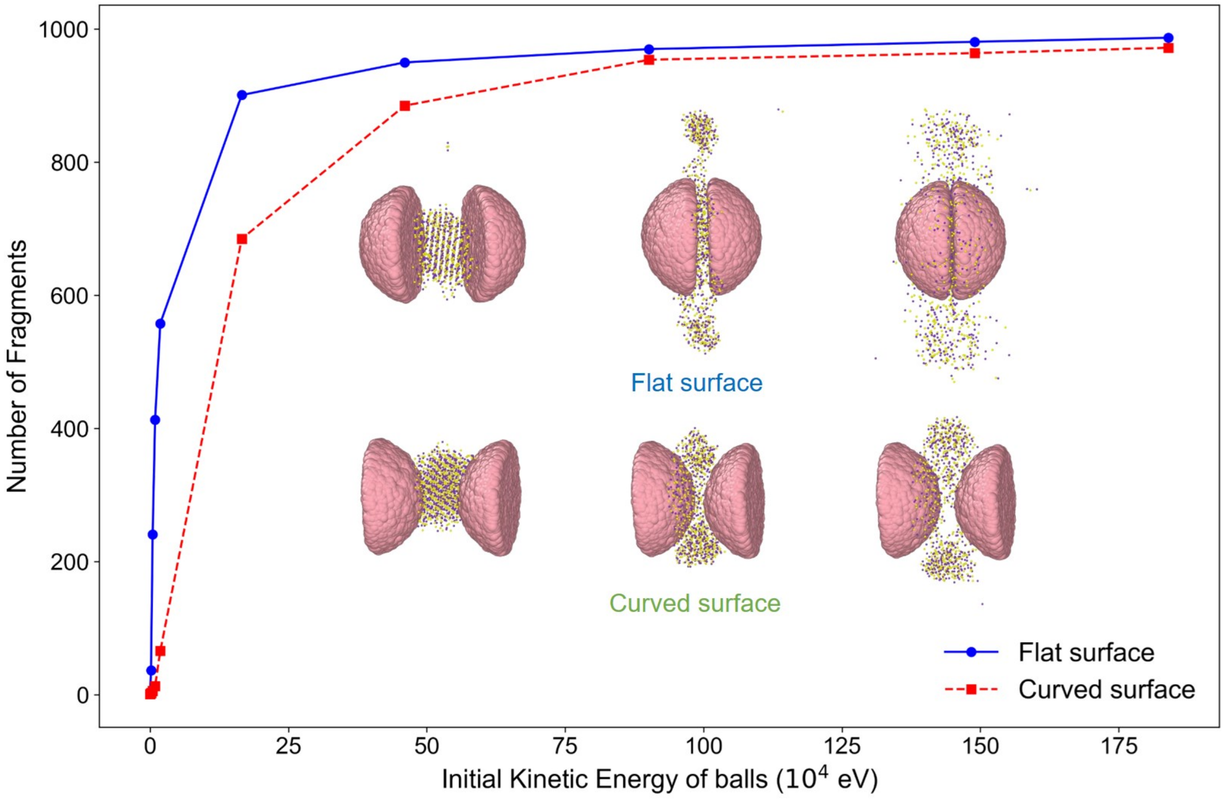

Figure S19: Number of fragments as a function of the kinetic energy of the milling balls for flat and curved impact surfaces of hemispherical milling ball designs. By a fragment we consider a collection of ions which have a neighbor within 3.9 Å (note that crystal KCl bond length is 3.15 Å).

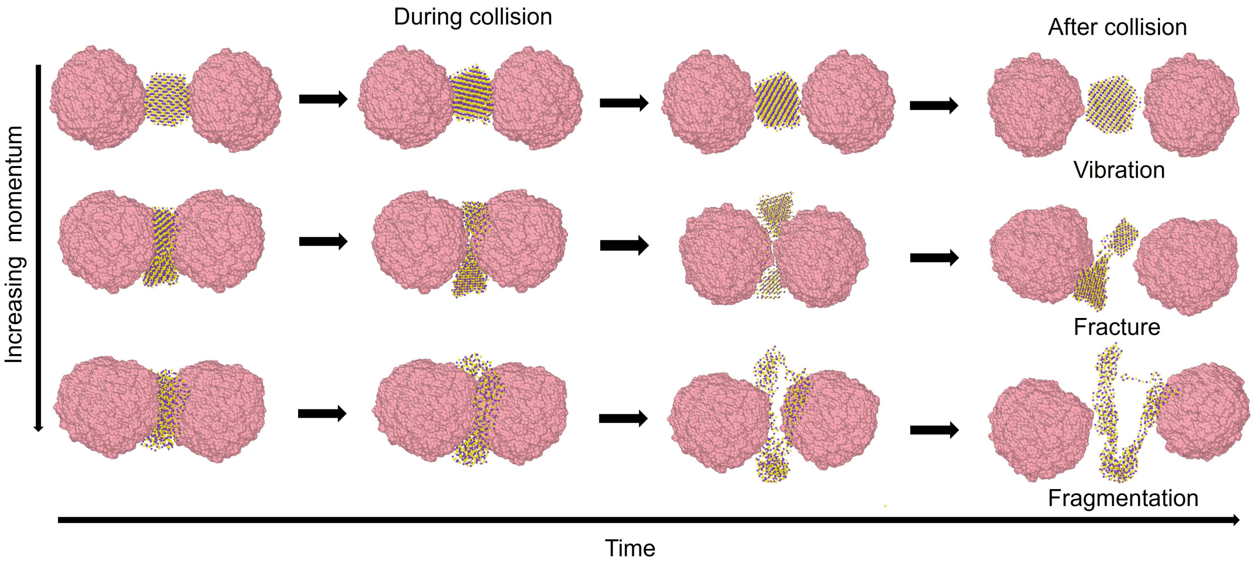

Figure S20: Snapshots depict the impact of two milling balls on the crystal over time, highlighting the effects of increasing ball momentum. The top row illustrates the lowest initial momentum, where in collision, the crystal undergoes vibrations without fracturing. As momentum increases (the second roq), the crystal breaks into a few fragments. At high absorbed energies (bottom row), full fragmentation occurs. The first three snapshots capture images during the collision, while the final column shows the milling balls rebounding after collision.

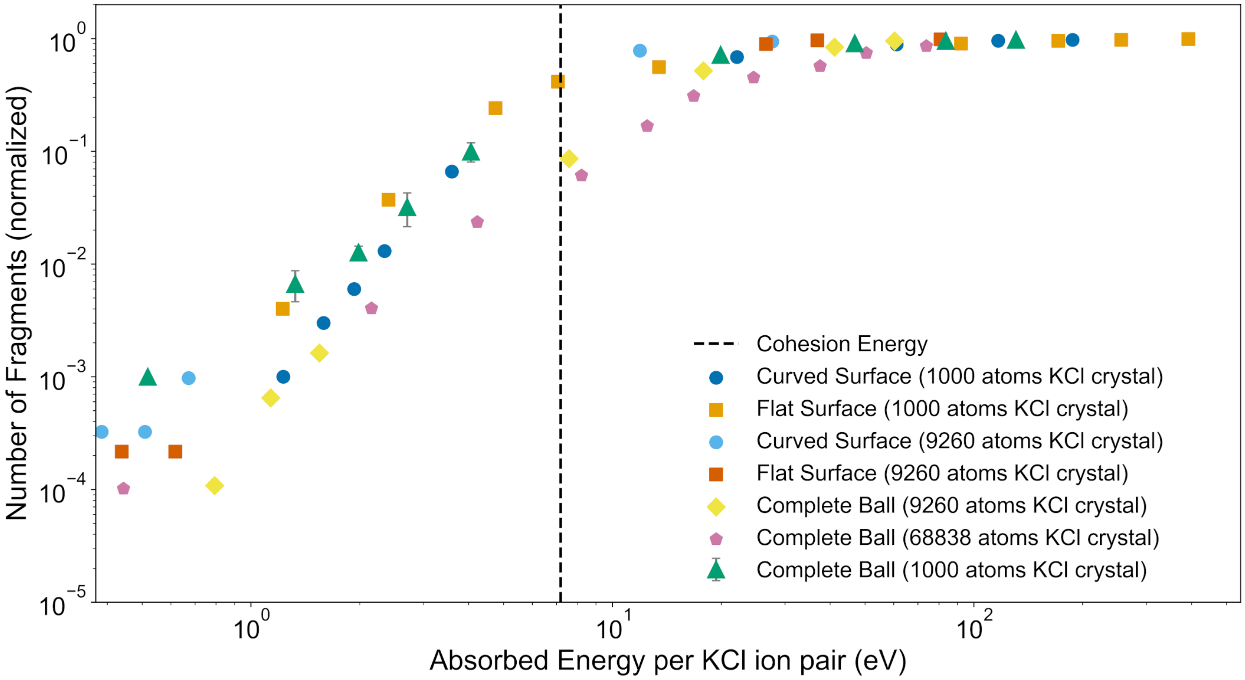

Figure S21: Comparison of fragmentation of KCl crystals with different geometries of the balls and with increasingly larger KCl crystals, normalised to the number of KCl ion pairs. The close overlap of different fragmentation curves demonstrate the insensitivity to crystal size and impact geometry and highlight the importance of the absorbed energy.

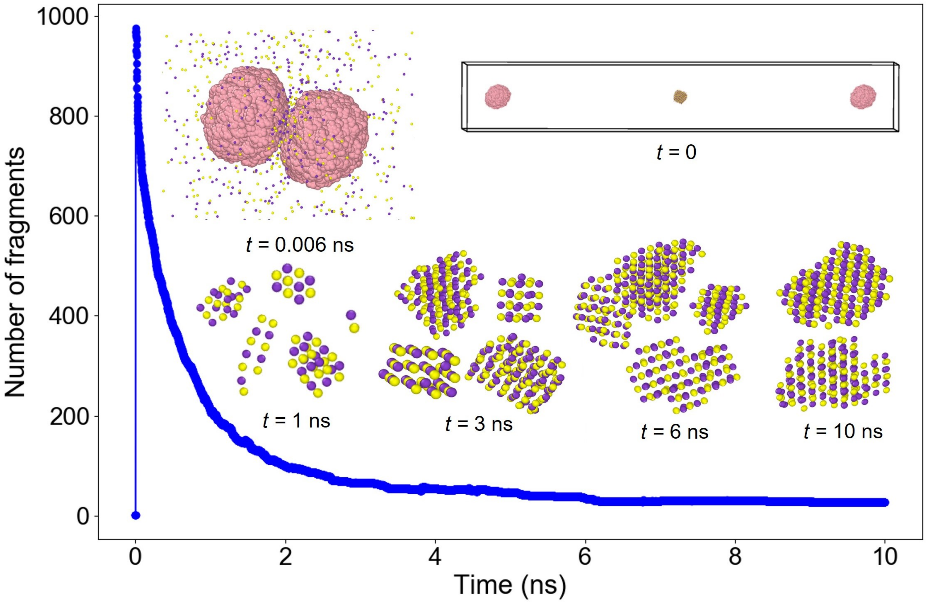

Figure S22: Coarsening of KCl crystal over time after collision with milling balls at an initial velocity of 10 nm/ps. By a fragment we consider a collection of ions which have a neighbor within 3.9 Å . The inset images capture the trajectory from t=0 to complete fragmentation of the KCl crystal at 0.006 ns, followed by formation of nanocrystals of FCC symmetry at 3 ns. As coarsening progresses, aided by the milling balls, new nanocrystals form, and existing nanocrystals increase in size and order, as seen at t = 6 and 10 ns.

S2.4 Simulations of dry complexation: 18c6 with KCl

S2.4.1 Simulation methods

To study the mechanochemical reaction between 18-crown-6 (18c6) and KCl crystal, the previously prepared balls and one of the KCl crystal were placed in a rectangular prism-shaped box with dimensions of ($x \times y \times z = 130 \times 20 \times 20$ nm^3), as described in the SI sections 2.3.1 and 2.3.2. Furthermore, a total of 500 18c6 molecules were uniformly distributed within the system. A short energy minimization run was performed to ensure no overlap of the existing species. This was followed by an MD run in the NVT ensemble, which was conducted for 5 ns at 375 K. A timestep of 0.1 fs and a time constant of 0.1 ps for the velocity-rescale thermostat was chosen for this equilibration.

During this run, the centers of mass of the balls and KCl crystal were constrained to maintain the head-on collision configuration, while 18c6 molecules were thermalised and allowed to move. This resulted in the formation of 18c6 droplets, which accumulated on the exposed crystal corners and the ball surfaces without absorption. This is evident by the fact that any displacement of the crystal or milling balls results in the separation of the droplet. For the KCl crystal, an independent NVT equilibration was performed, where the crystal was heated to 375 K (see SI section 2.3.1)

At the start of the milling simulations, the ions within the KCl crystal particles and 18c6 molecules were assigned velocities from a prior NVT equilibration. All atoms in the milling balls were assigned equal and opposite velocities of 10 nm/ps, directing them toward each other to ensure a three-body collision with the KCl crystal. A 0.1 fs timestep and a 2 ps time constant for the thermostat was used for the milling run with, which lasted 20 ns. The simulations were performed independently for 3 distinct crystal configurations.

All input files are available in Section: Milling KCl crystal and 18c6 of folder "Ball Milling Paper" in Zenodo - 10.5281/zenodo.13734954.

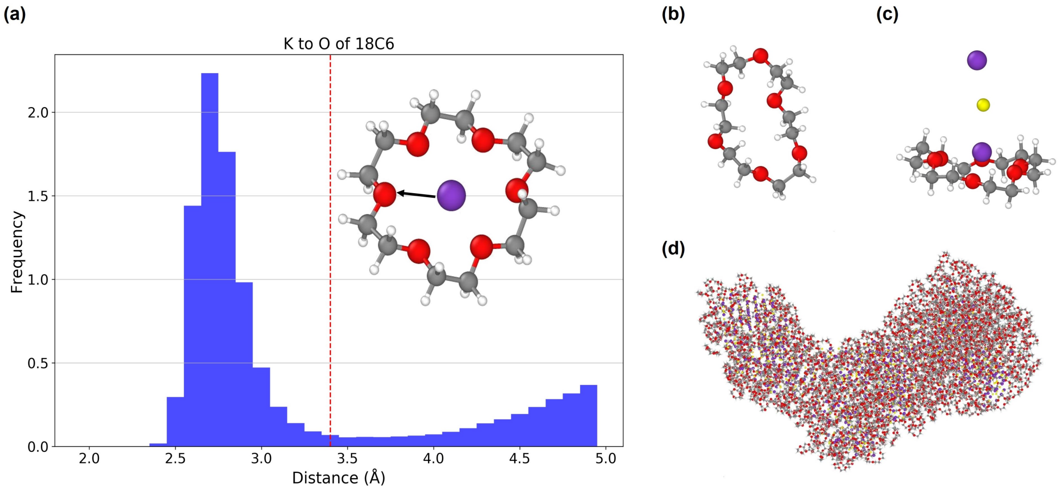

Figure S23: Analysis of characteristic structures. (a) Distribution of distances between potassium ions (purple ball) and the oxygen atoms (red) of the 18-crown-6. The inset showcases an example of a complex. Examples of (b) a freely roaming crown, (c) a small fragment group, and (d) a large agglomerate.

S2.4.2 Fragment analysis

In a mechanochemical environment under non-equilibrium conditions, tracking the evolution of species and monitoring their interactions is inherently challenging. To address this complexity and simplify analysis, it was necessary to group entities and define thresholds for most commonly interacting species.

For this purpose, we develop two Python scripts utilizing the MDAnalysis library to analyze molecular dynamics trajectories and identify different groups of entities in a simulation [22, 23]. It reads molecular trajectory data from a TPR (portable binary run input) file and an XTC (compressed trajectory) file and processes it frame by frame.

The first script complexcount.py counts the number of complexes, where a complex is defined as as a potassium ion (purple) coordinated by all six oxygen atoms of a single 18-crown-6 molecule (red and gray ring) within 3.4 Å. The threshold value of 3.4 is obtained by plotting the distance histogram between K^+ and oxygen atoms of 18c6 molecule as shown in Fig. S23a.

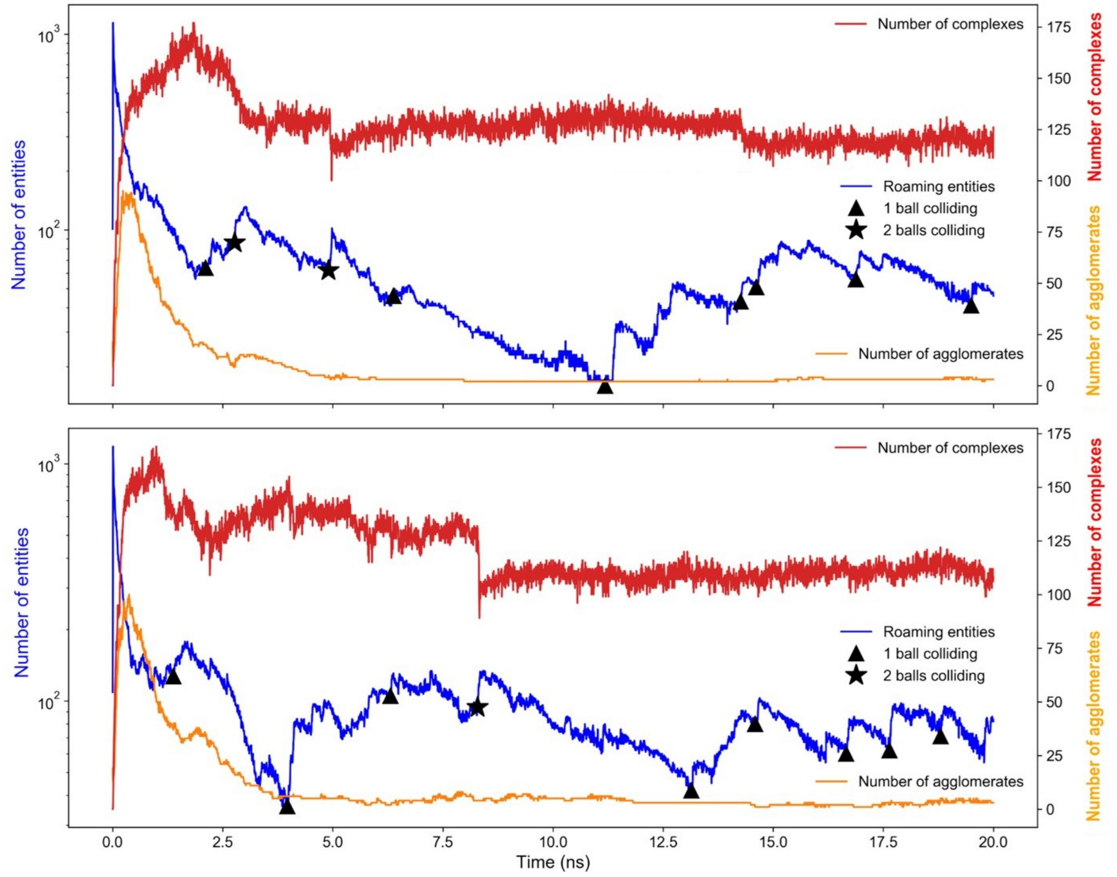

Figure S24: Simulation results for two distinct KCl crystal configuration of the mechanochemical reaction between KCl and 18c6. Time evolution of the number of complexes (18c6 containing K^+ in the cavity) (red), agglomerates (orange), and roaming entities that are not within an agglomerate (blue). Secondary collisions of a single ball with product are marked by black triangles whereas the simultaneous collisions of two balls with the product are represented by black stars.

The second script entities.py deals with grouping entities based on their number of neighbors in the following 3 categories :

- Freely roaming fragments: Isolated entities with no neighbors within 5 Å as shown in S23b.
- Small fragment groups: Entities with 1-3 neighbors identified using a cutoff of 3.4 Å for the neighborhood entities as shown in S23c.
- Number of large agglomerates: Aggregate of 5 or more entities as shown in S23d.

The three groups are mutually exclusive to each other. The scripts are available on :
`https://github.com/lvugrin/Ball_Milling_Paper.git`
under the folder `Cluster_Analysis`.

The simulations were performed independently for 3 distinct crystal configurations. The simulation results for 1 crystal configuration are shown in the main text Figure 3 while additional results are presented in Figure S24.

S2.4.3 Relaxation dynamics

The subsequent collisions between the balls and agglomerates, following the initial impact with the KCl crystal, are critical. These collisions occur at significantly lower ball kinetic energies compared to the first head-on impact. This is due to the gradual dissipation of energy as the system thermalizes. The collision of a single ball with agglomerates predominantly leads to mixing of the species and promotes coarsening, although occasionally they fracture small agglomerates (see Figure S25 a). However there are rare instances when an agglomerate is smashed by two milling balls which leads to the fracture of even big agglomerates (see Figure S25 b), reforming of complexes and a rise in the number of roaming entities.

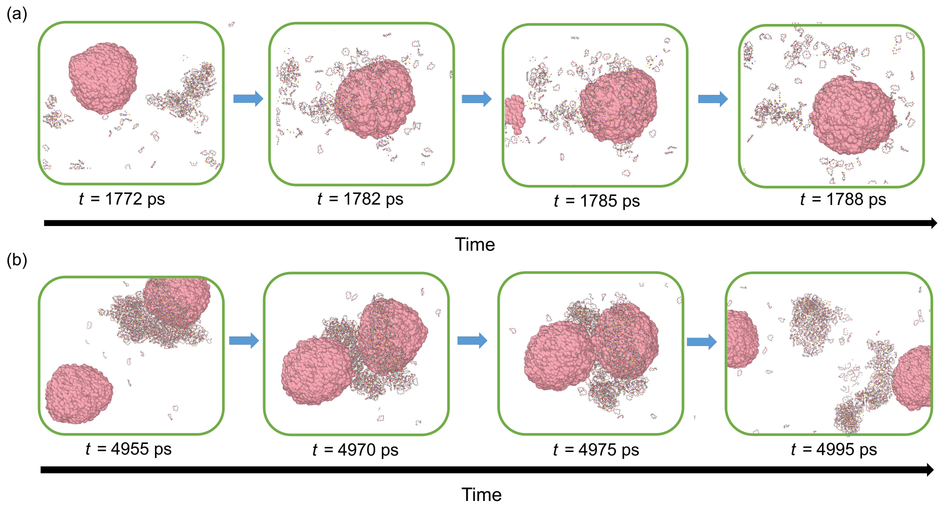

Figure S25: Secondary collisions. (a) A single ball undergoes a collision with a small agglomerate triggering its breakdown into smaller agglomerates while individual entities are also released. (b) Two balls collide with a large agglomerate resulting in a more extensive fragmentation compared to the one in the top row.

S2.5 Wet complexation of 18c6 with KCl

S2.5.1 Simulation methods

To investigate the role of H_2O in the mechanochemical reaction, we built two more systems with 200 and 2000 H_2O molecules. These systems were constructed identically as for dry complexation, with the KCl crystal placed centrally between two milling balls, and 500 18c6 molecules dispersed in the system. However this time, we also distribute either 200 or 2000 H_2O molecules throughout the box using the gmx solvate tool. A short energy minimization run was performed to ensure no overlap with the existing species. A MD run in the NVT ensemble was conducted for 5 ns at 375 K with the balls and KCl crystal frozen in their position, thermalising 18c6 and H_2O molecules. This produced a solution consisting of 18c6 and H_2O molecules in droplets. They also accumulated on the exposed crystal corners and the ball surfaces without absorption. A timestep of 0.1 fs and a time constant of 0.1 ps for thermostat was chosen for this equilibration.

The KCl crystal particles were assigned velocities from a prior NVT equilibration, where the crystal was independently heated to 375 K. At the start of the milling simulations, all atoms in the balls were assigned equal and opposite velocities of 10 nm/ps, directing them toward each other to ensure a three-body collision with the KCl crystal. The milling simulation was conducted with a 0.1 fs timestep and a 2 ps thermostat time constant over a duration of 20 ns. The simulations were performed independently for 3 distinct crystal configurations.

All input files are available in Zenodo archive 10.5281/zenodo.13734954, Section Milling KCl crystal, 18c6 and water.

S2.5.2 Fragment Analysis

We first generate and analyze the distribution of distances between specific pairs of species in the system as shown in Fig. S26, by developing a third script hydratedspecies.py The script is available on : `https://github.com/lvugrin/Ball_Milling_Paper.git` under the folder `Cluster_Analysis`.

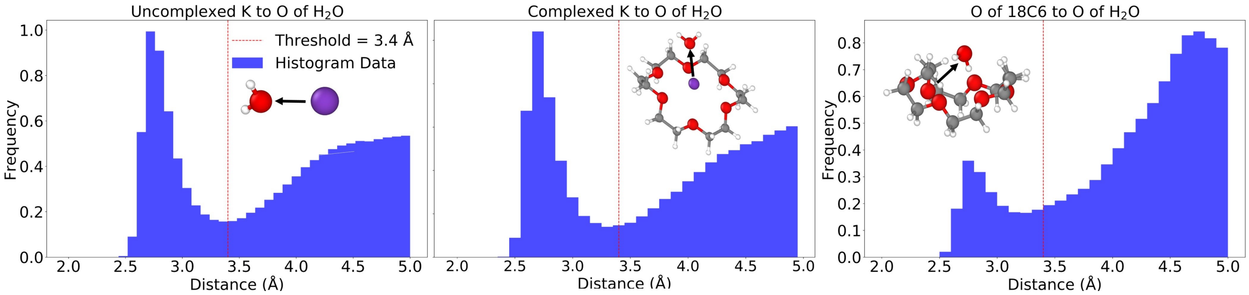

Figure S26: Distributions of distances between pairs of specific entities. The insets in each subplot provide visual representations of studied. (Left panel) separation between a water oxygen and a potassium ion out of the complex, (middle panel) distance of the water oxygen to the potassium within the complex and (right panel) distance between water oxygen and oxygen atoms of the 18-crown-6.

We find that the typical distance of a water molecule from a potassium ion or a crown is found to be 2.7 Å, with variations 0.2 Å. All distributions, however, have a clear minimum at 3.4 ± 0.1 Å, which is than taken as a threshold in a neighbor search algorithms. This threshold was incorporated in the script to calculate the number of hydrated and non hydrated K^+ ions, 18c6-K complexes, and 18c6 molecules, as shown in Fig. S27.

The time evolution of these species reveal that freely roaming K^+ (blue curve) basically vanishes from the system within a nanosecond after the collision, both in the system with high and low water content. When there is an abundance of water (left panel), long term dynamics demonstrates that due to the abundance of water, the likelyhood of hydrating a potassium is larger than the likelihood of finding a complex with the crown (green curve is above the orange curve in Fig. S27). Furthermore, during the slow equilibration (around 30 ns after collision), a steady growth of hydrated potassium on the expense of the number of complexes is found.

At the low water content (right panel in Fig. S27), the equilibrium is reached 15 ns after the collision when the number of complexes (orange curve) saturates above the generally low number of hydrated K^+ ions (green curve, note the logarithmic axis).

Finally, the fact that we can find the same characteristic species separations, allows us to use the methodology and scripts developed in the section S2.4.2 for the analysis of the number of fragments and the agglomerates in the studies of the relaxation dynamics in wet conditions.

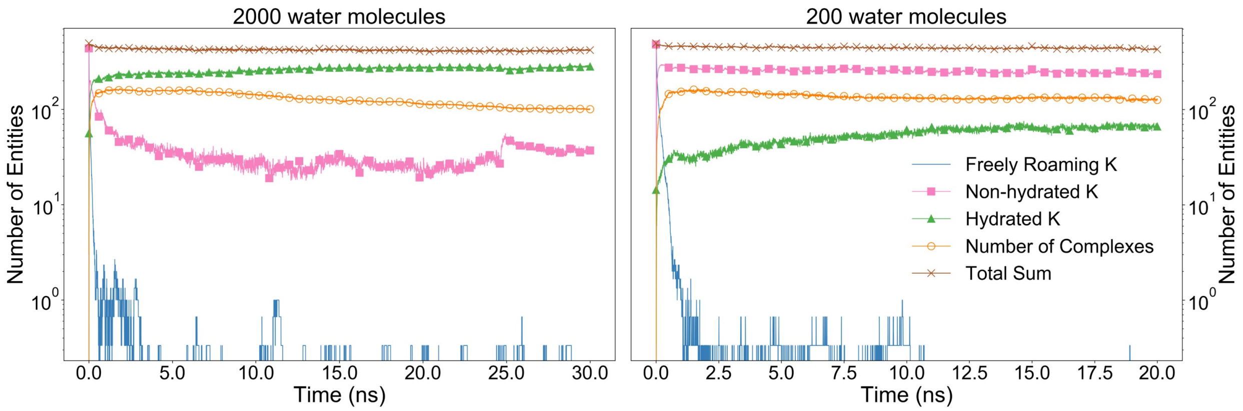

Figure S27: Time evolution of different environments of a K^+ ion in systems with 2000 water molecules (left panel, 30 ns run) and 200 water molecules (right panel), 20 ns run. Number of freely roaming K^+ (blue points), hydrated K^+ (green triangle), non-hydrated K^+ interacting with Cl^- ions (pink squares), 18c6-K^+ complexes (orange circles). For varification the total number of K^+ ions is also shown (brown crosses). Note that accomplishing steady state takes significantly longer in the system with the high water content.

S2.5.3 Relaxation Dynamics

We study the relaxation dynamics in wet conditions with low and high water content (i.e. 200 and 2000 water molecules in the system). The simulations are performed for three distinct crystal configurations, with results from each individual run presented in Figure S28 and as an average over these three runs are presented in the manuscript Figure 4a. A notable observation is that water

makes a largest fraction of freely roaming species both in low and high water content systems. However, In abundance of water, the concentration of freely roaming water is very similar to that of complexes, facilitating the competition for the potassium ions.

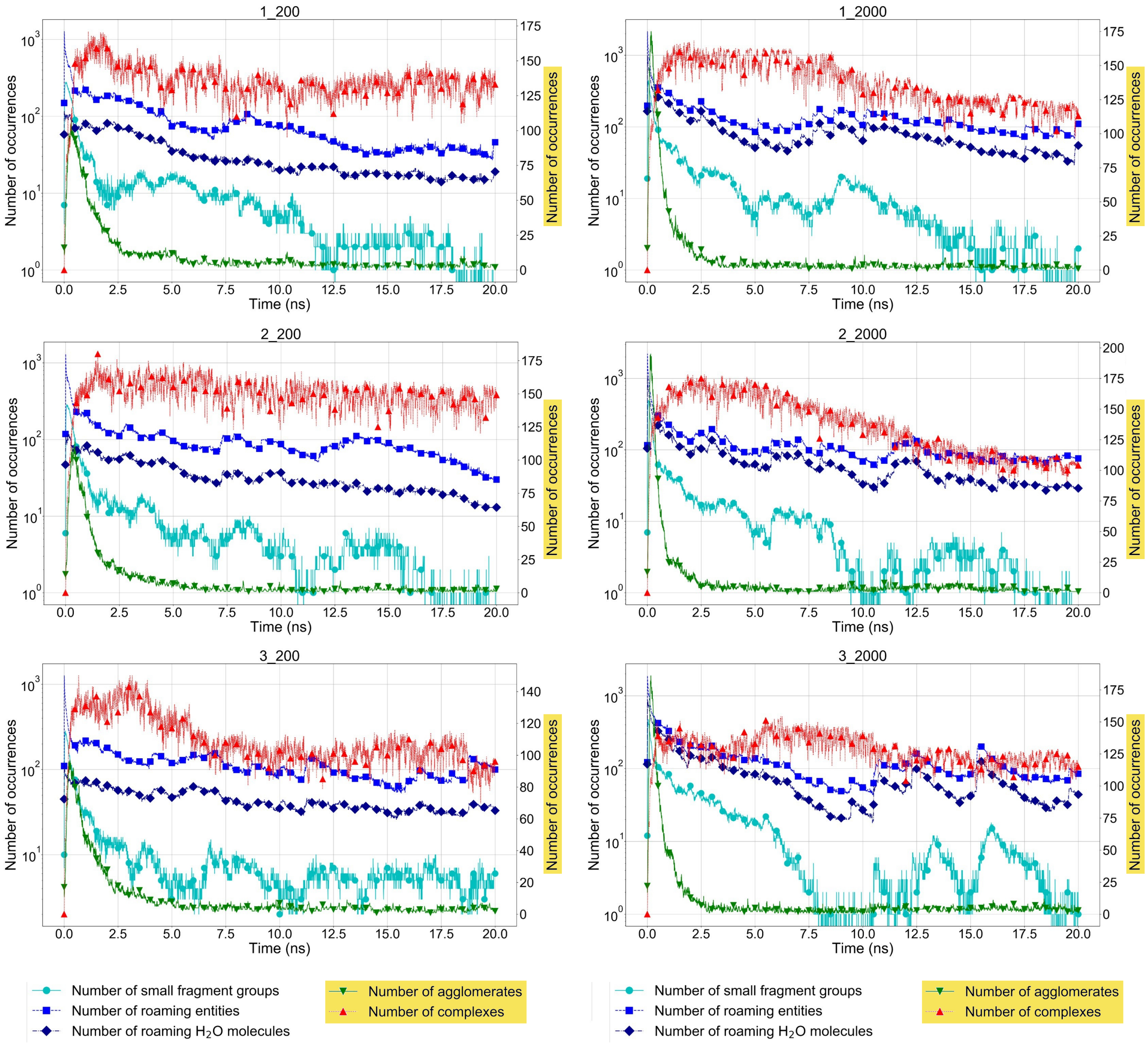

Figure S28: Fragmentation followed by complexation and formation of agglomerates for every run with three distinct initial crystal configuration. The plot titles are composed of two numbers: the first identifies the unique KCl crystal configuration (1 to 3), and the second indicates the number of H_2O molecules (200 or 2000). The number of complexes (red triangles) and agglomerates (green inverted triangles) are shown with the linear left axis. Freely roaming entities (blue squares), small fragment groups (cyan circles), and roaming H_2O molecules (black diamonds) are associated with the logarithmic right axis.

Like in previous simulations, we also find lower-energy collisions between milling balls and agglomerates during the coarsining process. These secondary collisions contribute to the mixing of species mixing and coarsening (as discussed in section S2.4.3), occasionally fracturing small clusters (S29a. Rare dual-ball impacts can shatter larger agglomerates, promoting complex reformation and increasing the probability to find freely roaming entities((S29b).

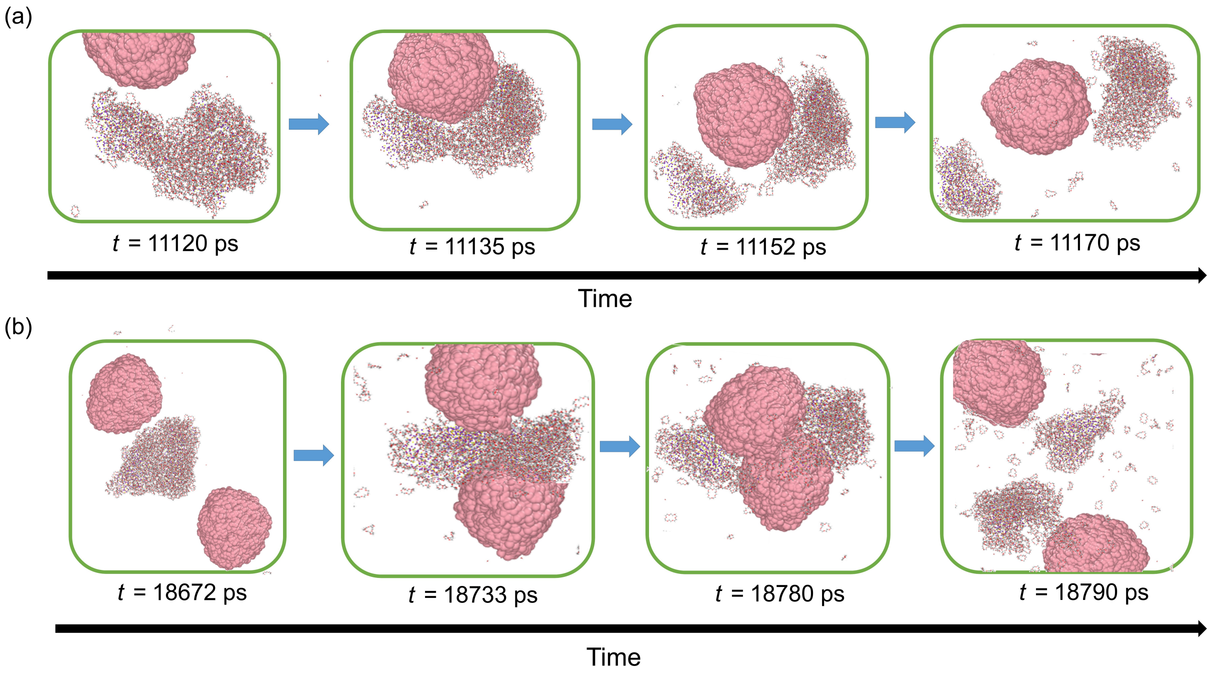

Figure S29: A sequence of events depicting the secondary collisions of balls with agglomerates during the milling simulation. (a) A single ball undergoes a collision with a small agglomerate triggering its breakdown. The agglomerate is fragmented into smaller agglomerates also while individual entities are released. (b) Two balls simultaneously collide with a large agglomerate resulting in a more extensive fragmentation compared to the one in the top row. This collision significantly enhanced the breakdown process, quickly reducing the agglomerate into smaller pieces and freeing more individual entities.

References

[1] S. Lukin, K. Užarević, I. Halasz, *Nat. Protoc.* **2021**, *16*, 3492.

[2] C. F. Macrae, I. Sovago, S. J. Cottrell, P. T. A. Galek, P. McCabe, E. Pidcock, M. Platings, G. P. Shields, J. S. Stevens, M. Towler, P. A. Wood, *J. Appl. Cryst.* **2020**, *53*, 226.

[3] J. Soler, E. Artacho, J. Gale, A. Garcia, J. Junquera, P. Ordejón, D. Sánchez-Portal, *Journal of Physics: Condensed Matter* **2002**, *14*.

[4] A. García, N. Papior, A. Akhtar, E. Artacho, V. Blum, E. Bosoni, P. Brandimarte, M. Brandbyge, J. I. Cerdá, F. Corsetti, R. Cuadrado, V. Dikan, J. Ferrer, J. Gale, P. García-Fernández, V. M. García-Suárez, S. García, G. Huhs, S. Illera, R. Korytár, P. Koval, I. Lebedeva, L. Lin, P. López-Tarifa, S. G. Mayo, S. Mohr, P. Ordejón, A. Postnikov, Y. Pouillon, M. Pruneda, R. Robles, D. Sánchez-Portal, J. M. Soler, R. Ullah, V. W.-z. Yu, J. Junquera, *The Journal of Chemical Physics* **2020**, *152*, 204108.

[5] J. P. Perdew, K. Burke, M. Ernzerhof, *Phys. Rev. Lett.* **1996**, *77*, 3865.

[6] S. Grimme, *Journal of Computational Chemistry* **2006**, *27*, 1787.

[7] NNIN/C Pseudopotential Virtual Vault, `https://nninc.cnf.cornell.edu/`, accessed: 2024-01-30.

[8] ATOM code for the generation of norm-conserving pseudopotentials, `https://docs.siesta-project.org/projects/atom`, accessed: 2024-02-11.

[9] H. Berendsen, D. van der Spoel, R. van Drunen, *Computer Physics Communications* **1995**, *91*, 43.

[10] E. Lindahl, B. Hess, D. van der Spoel, *J. Mol. Model.* **2001**, *7*, 306.

[11] J. Chandrasekhar, D. C. Spellmeyer, W. L. Jorgensen, *J. Am. Chem. Soc.* **1984**, *106*, 903.

[12] F. Berny, N. Muzet, R. Schurhammer, L. Troxler, G. Wipff, *MD Simulations on Ions and Ionophores at a Liquid-Liquid Interface: from Adsorption to Recognition*, pages 221–248, Springer Netherlands, Dordrecht **1999**.

[13] W. L. Jorgensen, D. S. Maxwell, J. Tirado-Rives, *J. Am. Chem. Soc.* **1996**, *118*, 11225.

[14] W. L. Jorgensen, J. Tirado-Rives, *Proceedings of the National Academy of Sciences* **2005**, *102*, 6665.

[15] L. S. Dodda, I. Cabeza de Vaca, J. Tirado-Rives, W. L. Jorgensen, *Nucleic Acids Research* **2017**, *45*, W331.

[16] L. S. Dodda, J. Z. Vilseck, J. Tirado-Rives, W. L. Jorgensen, *J. Phys. Chem. B* **2017**, *121*, 3864.

[17] H. J. C. Berendsen, J. R. Grigera, T. P. Straatsma, *J. Phys. Chem.* **1987**, *91*, 6269.

[18] A. Stukowski, *Modelling and Simulation in Materials Science and Engineering* **2010**, *18*, 015012.

[19] W. Humphrey, A. Dalke, K. Schulten, *Journal of Molecular Graphics* **1996**, *14*, 33.

[20] J. E. Stone, *An efficient library for parallel ray tracing and animation*, Master's thesis, University of Missouri–Rolla **1998**.

[21] K. Momma, F. Izumi, *Journal of Applied Crystallography* **2008**, *41*, 653.

[22] R. J. Gowers, M. Linke, J. Barnoud, T. J. E. Reddy, M. N. Melo, S. L. Seyler, J. Domanski, D. L. Dotson, S. Buchoux, I. M. Kenney, et al., MDAnalysis: A Python Package for the Rapid Analysis of Molecular Dynamics Simulations, Los Alamos National Laboratory (LANL), Los Alamos, NM (United States) **2019** .

[23] N. Michaud-Agrawal, E. Denning, T. Woolf, O. Beckstein, *Journal of Computational Chemistry* **2011**, *32*, 2319.

[24] M. J. Abraham, T. Murtola, R. Schulz, S. Páll, J. C. Smith, B. Hess, E. Lindahl, *SoftwareX* **2015**, *1-2*, 19.

[25] M. Abraham, A. Alekseenko, V. Basov, C. Bergh, E. Briand, A. Brown, M. Doijade, G. Fiorin, S. Fleischmann, S. Gorelov, G. Gouaillardet, A. Gray, M. E. Irrgang, F. Jalalypour, J. Jordan, C. Kutzner, J. A. Lemkul, M. Lundborg, P. Merz, V. Miletic, D. Morozov, J. Nabet, S. Pall, A. Pasquadibisceglie, M. Pellegrino, H. Santuz, R. Schulz, T. Shugaeva, A. Shvetsov, A. Villa, S. Wingbermuehle, B. Hess, E. Lindahl, GROMACS 2024.4 Manual **2024**.
